# Predicting the Grade of Prostate Cancer Based on a Biparametric MRI Radiomics Signature

**DOI:** 10.1155/2021/7830909

**Published:** 2021-12-23

**Authors:** Li Zhang, Xia Zhe, Min Tang, Jing Zhang, Jialiang Ren, Xiaoling Zhang, Longchao Li

**Affiliations:** ^1^Department of MRI, Shaanxi Provincial People's Hospital, Xi'an, Shaanxi 710000, China; ^2^Department of Radiology, Xijing Hospital, Fourth Military Medical University, 17 Changle Road, Xi'an, Shaanxi 710032, China; ^3^GE Health Care, Beijing 100000, China

## Abstract

**Purpose:**

This study aimed to investigate the value of biparametric magnetic resonance imaging (bp-MRI)-based radiomics signatures for the preoperative prediction of prostate cancer (PCa) grade compared with visual assessments by radiologists based on the Prostate Imaging Reporting and Data System Version 2.1 (PI-RADS V2.1) scores of multiparametric MRI (mp-MRI).

**Methods:**

This retrospective study included 142 consecutive patients with histologically confirmed PCa who were undergoing mp-MRI before surgery. MRI images were scored and evaluated by two independent radiologists using PI-RADS V2.1. The radiomics workflow was divided into five steps: (a) image selection and segmentation, (b) feature extraction, (c) feature selection, (d) model establishment, and (e) model evaluation. Three machine learning algorithms (random forest tree (RF), logistic regression, and support vector machine (SVM)) were constructed to differentiate high-grade from low-grade PCa. Receiver operating characteristic (ROC) analysis was used to compare the machine learning-based analysis of bp-MRI radiomics models with PI-RADS V2.1.

**Results:**

In all, 8 stable radiomics features out of 804 extracted features based on T2-weighted imaging (T2WI) and ADC sequences were selected. Radiomics signatures successfully categorized high-grade and low-grade PCa cases (*P* < 0.05) in both the training and test datasets. The radiomics model-based RF method (area under the curve, AUC: 0.982; 0.918), logistic regression (AUC: 0.886; 0.886), and SVM (AUC: 0.943; 0.913) in both the training and test cohorts had better diagnostic performance than PI-RADS V2.1 (AUC: 0.767; 0.813) when predicting PCa grade.

**Conclusions:**

The results of this clinical study indicate that machine learning-based analysis of bp-MRI radiomic models may be helpful for distinguishing high-grade and low-grade PCa that outperformed the PI-RADS V2.1 scores based on mp-MRI. The machine learning algorithm RF model was slightly better.

## 1. Introduction

Prostate cancer (PCa) is the most common malignancy in men [1]. Based on the Gleason score (GS), PCa is categorized as low-grade or high-grade, for which the treatment strategies differ greatly [2]. For instance, patients with high-grade PCa need radical prostatectomy or radiation therapy, whereas patients with low-grade PCa might be candidates for active surveillance [3–6]. Therefore, a preoperatively accurate prediction of the grade of PCa is critical for treatment decision-making.

Currently, biopsy is the reference standard for preoperatively identifying the grade of PCa [7]. However, this procedure has been shown to be susceptible to overdetection of low-grade and underdiagnosis of high-grade PCa [8]. Therefore, developing a noninvasive and accurate approach for the preoperative prediction of grade is desirable.

Multiparametric (Mp)-MRI has been recognized as a complementary tool for the detection and assessment of PCa. Radiologists use the Prostate Imaging Reporting and Data System Version 2.1 (PI-RADS V2.1) to detect clinically significant PCa [9]. Nevertheless, mp-MRI interpretation is challenging and prone to inter- and intrareader variability among expert radiologists [8].

According to the literature, machine learning-based mp-MRI radiomics provide an objective tool and have been shown to be helpful in assessing the grade of PCa [10–12]. In contrast, dynamic contrast enhancement (DCE) is a time-consuming process with additional costs of contrast agents [13–15]. Additionally, a meta-analysis reported that DCE-MRI cannot be used to predict GS in PCa [16].

To our knowledge, few studies have predicted grades using biparameter (bp)-MRI (noncontrast agent) with radiomics [17]. However, machine learning algorithms are relatively simple, and the results of radiomics have not been compared with the traditional PI-RADSV2.1 approach. Thus, the aim of this study is twofold. First, we used three machine learning methods of radiomics based on bp-MRI, including logistic regression, random forests (RF), and support vector machines (SVM), to preoperatively predict PCa grade. A second purpose was to compare these classifying capabilities to the visual assessments of the radiologists based on mp-MRI of the PI-RADS V2.1 protocol.

## 2. Materials and Methods

The local institutional review board approved this retrospective cohort study and waived the requirement for written informed consent.

### 2.1. Patients

Between January 2017 and November 2020, a total of 166 consecutive patients who underwent prostate mp-MRI examinations with histologically confirmed PCa were included in this study. The exclusion criteria were as follows: (a) patients who received prior treatment including hormonal irradiation before the MRI scans (*n* = 21); (b) poor quality of the MRI images due to severe susceptibility artifacts or respiratory motion artifacts (*n* = 2); and (c) incomplete clinical data (*n* = 1).

Ultimately, 142 patients with PCa were enrolled in this study. For patients with multiple tumor sites, the site with the largest burden (namely, the largest size or the highest GS) was reported in the document. Therefore, in this study, only one tumor site from each patient was used for analysis [17].

**Figure 1:**
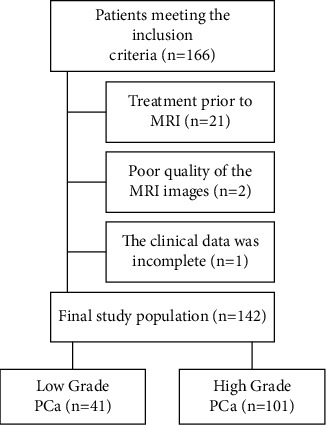
Flow diagram of patient recruitment.

According to recent radiomics studies [18], these enrolled patients were randomly divided into a training cohort (*n* = 98) and a test cohort (*n* = 44) using computer-generated random numbers at a 7 : 3 ratio. Flow diagram of patient recruitment is shown in Figure 1.

### 2.2. MRI Protocol and Feature Extraction

All images were acquired using a 3.0 TMR scanner (Achieva TX, Philips Healthcare, and the Netherlands) with a 16-channel body phased array coil. A standard mp-MRI protocol included sagittal T2WI, axial T2WI, diffusion-weighted imaging (DWI) (b values of 0 and 1000, 2000 sec/mm^2^), and DCE. Apparent diffusion coefficient (ADC) maps were automatically reconstructed on a designated workstation. The detailed acquisition parameters of the MRI sequences are shown in Table 1.

**Figure 2:**
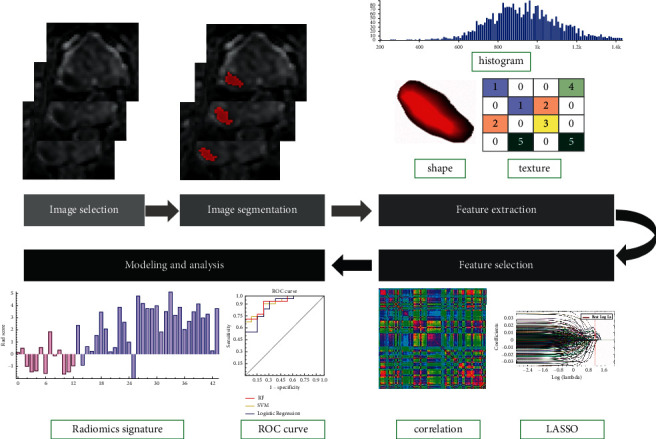
Radiomics workflow and study flowchart.

Both the ADC and T2WI images were retrieved from a picture archiving and communication system (PACS, Huahai). The MRI images were loaded into ITK-SNAP software (version 3.4.0; https://www.itksnap.org) semiautomatically, and a three-dimensional volume of interest (VOI) that covered the whole tumor was delineated on both the axial ADC and T2WI images on each slice segmented by one radiologist (radiologist C, Z. L., with 10 years of experience in prostate MRI). The procedure of VOI is shown in Figure 2.

Texture extraction was performed using artificial intelligence kit (A.K., v. 3.2.1, GE Healthcare) software. In total, 804 imaging features were extracted from each VOI, including first-order statistics, histogram features, second-order textures, and form factor (shape) parameters.

Then, interclass correlation coefficients (ICCs) were used to assess the interobserver reproducibility of radiomic feature extraction. Approximately 30 randomly chosen images were obtained by a senior radiologist (radiologist D, Z. X. L., with 20 years of experience in prostate MRI) for VOI segmentation.

### 2.3. Feature Selection, Radiomics Signature Construction, and Model Training

Not all the extracted features were useful for differential diagnosis. The abovementioned features were selected through least absolute shrinkage selection operator (LASSO) regression analysis with a 10-fold cross-validation and Spearman correlation (threshold 0.9) coefficient for dimensionality reduction and feature selection to optimize the size of a feature set and to keep features independent.

**Figure 3:**
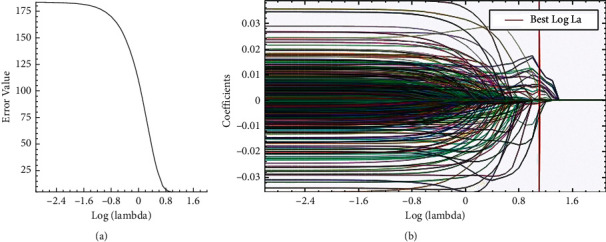
The process of feature selection using the LASSO algorithm. (a) The optimal tuning parameter (lambda) in the LASSO model was selected using 10-fold cross-validation and the 1 standard error rule. (b) LASSO coefficient profiles of the 26 features. The vertical line was drawn according to the 10-fold cross-validation in (a). LASSO, least absolute shrinkage selection operator.

Finally, 8 features were extracted from the T2WI and ADC images. The process of feature selection using the LASSO algorithm is shown in Figure 3.

**Figure 4:**
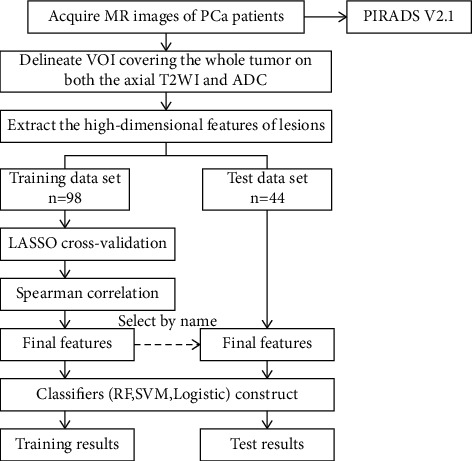
The process of the study.

The goal of the machine learning routine was to construct a predictive model to discriminate between the two classes: low-grade and high-grade PCa. Three algorithms (RF, logistic regression, and SVM) were proposed as classifiers [19–21]. They can be used to find worthwhile features and remove relatively insignificant features to achieve higher classification performance [22–26]. The model assessed in the training dataset was applied to the test cohort. The radiomics workflow is presented in Figure 2, and the process is outlined in Figure 4.

### 2.4. PI-RADS V2.1 Evaluation

All images were networked to a communication workstation (Huahai Medical Imaging PACS, Xi'an, China) and Philips workspaces. According to the PI-RADS V2.1 guideline, two independent radiologists with different levels of experience (radiologist A, Z. J., with 3 years of experience, and radiologist B, T. M., with 10 years of experience in prostate MRI diagnosis), who were blinded to the initial mp-MRI imaging reports, clinical data, and histopathology, scored the examinations. In addition, the two radiologists did not previously participate in the process of VOI delineation. The PI-RADS V 2.1 score was independently recorded by radiologists using a score of 1–5 for T2WI, a score of 1–5 for DWI, a “+” or “−” for DCE, and an overall PI-RADS assessment category. When multiple doubtful lesions appeared in the same patient, only the most suspicious lesion with the maximum volume was scored and recorded [27]. Lesions graded as having a PI-RADS ≥ 4 were considered positive for high-grade PCa, and lesions with a score ≤3 were considered negative for low-grade PCa. The end performance for both radiologist and the radiomics model was computed based on patient-level classification. The detailed evaluation items of PI-RADS V2.1 scale (Supplement Tables 1–5) are uploaded in the supplementary materials.

### 2.5. Statistical Analysis

A kappa test was used to assess the inter-reader agreement of the PI-RADS V2.1 scores obtained by the two radiologists. Kappa values < 0.20 indicated poor agreement, 0.21–0.40 indicated fair agreement, 0.41–0.60 indicated moderate agreement, 0.61–0.80 indicated good agreement, and ≥0.81 indicated excellent agreement [28]. The ICC was used to evaluate the interobserver reproducibility of the extracted radiomic features. An ICC score greater than 0.75 indicates good agreement of the feature extraction. The training and test cohorts were used to verify the diagnostic performance of the prediction models with RF, logistic regression, and SVM. The predictive ability of the radiomics signature models based on bp-MRI vs. mp-MRI of the PI-RADS V2.1 score in identifying low-grade and high-grade PCa was analyzed based on receiver operating characteristic (ROC) curves. The performance of radiomics models was compared by the DeLong test [29]. The areas under the curve (AUC), sensitivity, specificity, positive likelihood ratio (LR+), and negative likelihood ratio (LR-) were derived in both the training and test cohorts. High-grade tumors were considered the positive class.

*P* < 0.05 indicates statistical significance. The statistical analysis was performed with the Statistical Package for the Social Sciences (SPSS, https://www.ibm.com/products/spss-statistics), MedCalc software, and A.K. software (mentioned above).

## 3. Results

### 3.1. Clinicopathologic Characteristics of Patients

The baseline characteristics of selected patients whose data were classified into the training and test cohort are summarized in Table 2. There were no significant differences in terms of age or PSA density (PSAD) between the two groups (all *P* > 0.05). Significance differences between high-grade and low-grade PCa were found in PSA level in the training and test cohorts (all *P* < 0.05). The difference in location was statistically significant in the training cohort (*P* < 0.05).

### 3.2. Radiomics Signature Construction

The reliability of the extracted radiomic features in terms of ICC for all features of ADC and T2WI images was quantified, with mean ICC values of 0.919 (95% CI: 0.836–0.960) and 0.963 (95% CI: 0.925–0.982), respectively.

A total of 804 quantitative features were extracted from the VOI of each of the MRI series and their corresponding filtered results.

In the current study, 8 features were obtained from bp-MRI (constructed from 8 optimal feature sets selected from 804 features combined by T2WI and ADC sequences). The process of feature selection using the selection step is shown in Table 3.

A machine learning-based analysis of the bp-MRI radiomics signatures was established based on T2WI combined with ADC images for (a) RF; (b) logistic regression; and (c) SVM model.

### 3.3. Predictive Ability of the Radiomics Signatures (Models)

**Figure 5:**
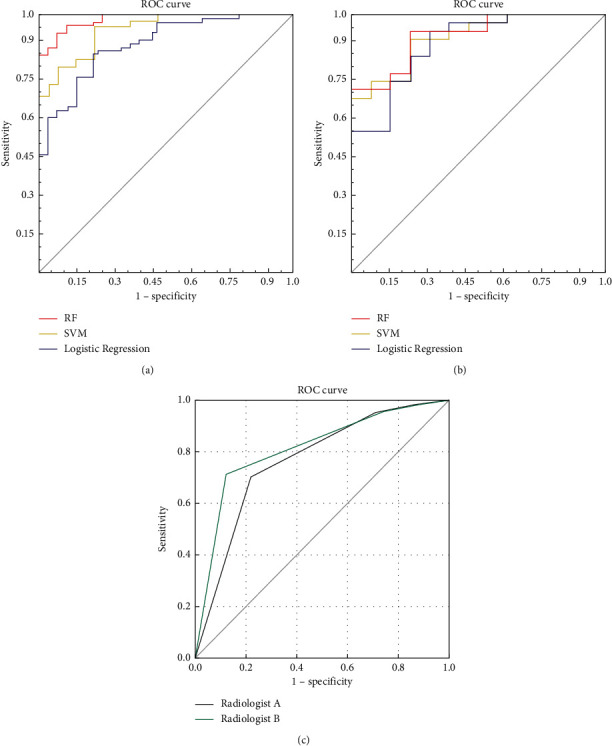
ROC curves of the radiomics signature in the training (a) and test (b) cohorts. ROC curves for the radiomics-based ADC and T2WI model and PI-RADS (c) score performance in distinguishing low- vs. high-grade PCa in the training and test groups. RF, random forest tree; SVM, support vector machine. Predictive ability of the PI-RADS V 2.1 model.

The results show that the differential diagnostic model established using the RF method (AUC: 0.982; 0.918) performed better than logistic regression analysis (AUC: 0.886; 0.886) and SVM (AUC: 0.943; 0.913) in both the training and test cohorts. The DeLong test showed that the RF method presented a better AUC than logistic regression analysis in training cohorts (*P* < 0.05). The ROC curves of the radiomic signatures for discriminating performance in both the training and test cohorts are shown in Figure 5. The predictive ability (AUC, sensitivity, specificity, LR+, and LR−) of the radiomics model is shown in Table 4 for the training and test groups (see Supplementary Materials for the original radiomics data).

The kappa coefficient of the two radiologists was 0.7, which indicated good agreement. Based on a PI-RADS V2.1 with a score of 5 as the cutoff value, the AUCs of the two radiologists' diagnosis performances were 0.767 and 0.813, respectively, which were lower than those of the radiomics feature classifiers in classifying high-grade and low-grade PCa (Figure 5 and Table 4).  Table 5 lists the results of the evaluations of all images by the two radiologists. The scores for each case are based on the PI-RADS V2.1 scale of radiologists A and B, which are uploaded in the supplementary materials (Supplement Table 6).

## 4. Discussion

Preoperative prediction of PCa grade is important for clinical decision-making. Pathological biopsy is a popular tool for the estimation of PCa grade invasiveness, but it has various complications that limit its clinical use.

In this study, we described a radiomics signature using noncontrast-enhanced MRI images, which is a new noninvasive method with good diagnostic performance. Our results showed that the machine learning-based analysis of bp-MRI-based radiomics models for distinguishing low-grade from high-grade PCa provided higher accuracy and sensitivity than visual assessments by radiologists based on the PI-RADS V2.1 mp-MRI score. This indicates that T2WI or ADC images could reflect the heterogeneity of tumors and that radiomic features can be used to distinguish small signal differences in prostate tumors. The present study also found that selecting the appropriate machine learning algorithms may help improve model stability and prediction performance. The results of our study show that the radiomics-based RF machine-learning models, which incorporated 8 selected features, have better discrimination performance than other models (SVM and logistic regression) for differentiating low-grade from high-grade PCa (AUC of the training cohort: 0.982; AUC of the test cohort: 0.918) in both the training and test cohorts.

Some studies suggest the advantages of logistic regression models in aggressive PCa [30, 31]. Generally, logistic regression, RF, and SVM algorithms are all suitable for model construction with small sample sizes and binary variables [30–34]. However, for bp-MRI (ADC combined with T2WI), the RF algorithm is more recommended for use with our small sample result.

We also assessed the predictive values of mp-MRI based on PI-RADS V2.1. In our study, the predicted probabilities of the radiomics features outperformed subjective evaluation by the human group.

Previous studies have evaluated the performance of MRI radiomic-based models for predicting aggressive PCa. A study by Parra et al. explored DCE and ADC-feature radiomics models for predicting clinically significant PCa with an AUC of 0.82 [35]. Ma S et al. reported that an mp-MRI radiomics signature that outperformed the radiologists' visual assessments in predicting extracapsular extension was developed and validated [36]. Niu et al. reported that a texture analysis-based model that includes bp-MRI can be used for identifying high-grade PCa and that specific parameters extracted from texture analysis may be additional tools for assessing tumor aggressiveness [37]. Min et al. established an mp-MRI-based radiomics model for discriminating between clinically significant PCa and clinically insignificant PCa with an AUC of 0.823 [34]. However, these research methods only extract a few texture features or only use one machine learning model and are not compared with the traditional PI-RADS V2.1 score, and the feedback information is relatively limited. In the current study, three classifiers were utilized and compared with the PI-RADS V2.1 score; then, the most effective features were selected for the construction of the three prediction models.

bp-MRI allows for accurate detection and localization of suspicious PCa, in addition to a reduction of the time required to complete the study and lower costs without using gadolinium [38]. Moreover, potential risks related to the use of gadolinium-based contrast media, such as nephrogenic systemic fibrosis, renal failure, and accumulation of gadolinium in the brain, would be reduced [39]. In general, T2WI and ADC are the most common sequences selected by investigators for radiomics research [32, 40–42]. Therefore, we chose to derive the radiomic features from T2WI and ADC only for this study. In addition, using too many sequences affects clinical application because of time-consuming and laborious image segmentation, so it is more important to choose a valuable sequence. Therefore, we conclude that mp-MRI-based radiomics may contribute to differentiating low-grade from high-grade PCa and to risk stratification without the need for additional MRI sequences such as DCE.

High-grade PCa with more heterogeneous tissue conditions than low-grade PCa tumors exhibited on the spatial distribution of voxel intensities and radiomics could provide more information to distinguish cancers that are challenging for the PI-RADS V2.1 score [42, 43]. In addition, the clinical application of PI-RADS V2.1 is subjective and highly reader-dependent due to its natural feature of strictly fixed discrete categories. Moreover, PI-RADS V2.1 requires a DCE sequence and has the disadvantage of achieving quantitative criteria that favor diagnostic performance. In this study, we proposed automatic machine learning approaches to address these challenges and employed a well-recognized method, RF, leading to robust classifier performance without using gadolinium. RF is an ensemble algorithm with high accuracy and tolerance [44]. Its greatest advantage is that it can predict multivariate data and analyze complex nonlinear relationships [23].

However, there were also some limitations in this study. First, it was a retrospective single-center study, and the sample size was relatively small. Prospective multiple-center studies are needed in the future, and other external validation cohorts should also be included to test the reproducibility of future studies. Second, although a three-dimensional VOI was used, the deviation caused by subjective factors is inevitable when the VOI is manually drawn. We manually segmented and delineated the VOIs with the two radiologists in consensus, trying our best to reduce the deviation. Further studies should adopt semiautomatic segmentation. Third, some pathological results were proven by systematic combined targeted biopsy and lacked whole-mount serial section. Although whole-mount histopathology is considered the reference standard, it is unreasonable to expect that all of our cases would undergo radical prostatectomy, especially for low-grade PCa patients. Additionally, only including patients who underwent radical prostatectomy would lead to selection bias.

## 5. Conclusion

In summary, our study shows that machine learning-based analysis of the bp-MRI radiomic model presented superior diagnostic performance to traditional PI-RADS V2.1 scores for predicting the histological grade of PCa. The machine learning algorithm RF model was slightly increased. It was found that the algorithm assists radiologists in reporting tumor grade and facilitates clinical decision-making for the management of PCa.

## Figures and Tables

**Table 1:** The detailed acquisition parameters of the MRI sequences.

Parameters	Axial T2WI	DWI (*b* = 50, 1000, and 2000) (sec/mm^2^)	DCE
Field of view (mm)	200 × 200	220 × 220	260 × 260
Acquisition matrix	372 × 363	88 × 82	216 × 217
Repetition time (ms)	3020	3960	3.3
Echo time (ms)	100	86	1.59
Flip angle (degree)	90	90	10
Section thickness (mm), no gaps	3	3	2
Image reconstruction matrix (pixel)	339 × 339	160 × 160	288 × 288
Reconstruction voxel imaging resolution (mm/pixel)	0.34 × 0.34 × 4	1.63 × 1.63 × 4	0.9 × 0.9 × 2

Mp-MRI, multiparametric MRI; T2WI, T2-weighting imaging; DWI, diffusion weighted imaging; DCE, dynamic contrast-enhanced.

**Table 2:** The baseline characteristics of selected patients in the training and test cohorts.

Characteristics	Training cohort (*n* = 98)			Test cohort (*n* = 44)		
	High-grade PCa (*n* = 70)	Low-grade PCa (*n* = 28)	*P* value	High-grade PCa (*n* = 31)	Low-grade PCa (*n* = 13)	*P* value
Mean age (y)	73.19 ± 8.56	72.11 ± 7.58	0.562	74.77 ± 8.245	72.46 ± 8.353	0.403
Median PSA (ng/ml)	16.47 (IQR:8.21–61.78)	8.28 (IQR:6.36–14.03)	<0.05	15.3481 (IQR 8.28–62.1)	8.81 (IQR:6.27–14.41)	<0.05
Median PSAD (ng/mL/g)	0.15 (IQR:0.08–0.17)	0.15 (IQR:0.07–0.14)	0.76	0.20 (IQR:0.08–0.28)	0.18 (IQR:0.11–0.21)	0.7
Location						
PZ	25	13	<0.05	11	8	0.11
TZ	9	11		3	4	
PZ and TZ	36	4		17	1	

Date are mean ± SD. IQR, interquartile range; TZ, transition zone; PZ, peripheral zone; PSAD, PSA density.

**Table 3:** The process of feature selection using the selection step.

Lasso: cross validation	Spearman
“Min intensity”	“Min intensity”
“Histogram entropy”	“Histogram entropy”
“Correlation_AllDirection_offset1_SD”	“Correlation_AllDirection_offset1_SD”
“GLCMEntropy_AllDirection_offset1”	“GLCMEntropy_AllDirection_offset1”
“GLCMEntropy_AllDirection_offset4”	“GLCMEntropy_angle0_offset7”
“GLCMEntropy_AllDirection_offset7”	“Sum average”
“GLCMEntropy_angle0_offset1”	“HighGreyLevelRunEmphasis_AllDirection_offset4_SD”
“GLCMEntropy_angle0_offset4”	“Elongation”
“GLCMEntropy_angle0_offset7”	
“GLCMEntropy_angle135_offset1”	
“GLCMEntropy_angle135_offset4”	
“GLCMEntropy_angle135_offset7”	
“GLCMEntropy_angle45_offset1”	
“GLCMEntropy_angle45_offset4”	
“GLCMEntropy_angle90_offset1”	
“GLCMEntropy_angle90_offset4”	
“GLCMEntropy_angle90_offset7”	
“Hara entroy”	
“Sum average”	
“Sum entropy”	
“ShortRunHighGreyLevelEmphasis_AllDirection_offset1”	
“ShortRunHighGreyLevelEmphasis_angle0_offset1”	
“ShortRunHighGreyLevelEmphasis_angle0_offset7”	
“ShortRunHighGreyLevelEmphasis_angle45_offset1”	
“HighGreyLevelRunEmphasis_AllDirection_offset4_SD”	
“Elongation”	

LASSO, least absolute shrinkage selection operator.

**Table 4:** Discrimination for differentiating low-grade and high-grade PCa in the training and test group and PI-RADS V2.1 scores.

Model	Machine learning	AUC	Accuracy	Sensitivity	Specificity	LR+	LR−
T2WI combine ADC (training)	RF	0.982	0.857	0.92	0.714	3.2168	0.112
	Logistic regression	0.886	0.847	0.968	0.538	2.0952	0.0595
	SVM	0.943	0.857	0.957	0.621	2.5251	0.0692

T2WI combine ADC (test)	RF	0.918	0.795	0.935	0.462	1.7379	0.141
	Logistic regression	0.886	0.841	0.968	0.538	2.0952	0.06
	SVM	0.913	0.841	0.935	0.615	2.4286	0.106

PI-RADS V2.1	Reader 1	0.767	0.725	0.703	0.78	3.1955	0.381
	Reader 2	0.813	0.76	0.713	0.878	5.8443	0.327

ADC, apparent diffusion coefficient; T2WI, T2-weighting imaging; AUC, area under the curve; RF, random forest tree; SVM, support vector machine; LR+, positive likelihood ratio; LR−, negative likelihood ratio; PI-RADS V2.1, prostate imaging reporting and data system version 2.1.

**Table 5:** Performance of each of the radiologists in the PI-RADS V2.1 scoring of the high-grade PCa group and the low-grade PCa group.

PI-RADS V2.1scores	High-grade PCa		Low-grade PCa	
	Radiologist A	Radiologist B	Radiologist A	Radiologist B
1	1	0	3	0
2	1	2	3	5
3	3	3	6	5
4	25	25	20	26
5	71	72	9	5
Total	101	101	41	41

PI-RADS V2.1, prostate imaging reporting and data system version 2.1; PCa, prostate cancer.

## Data Availability

The data used to support the findings of this study are included within the Supplementary Information file.

## Supplementary Materials

Supplementary Materials The supplementary file contains the supplementary information about the original data for radiomics (Supplementary Materials and Supplement Tables 1–6).
