# Supplementary material for: Predicting the Grade of Prostate Cancer Based on a Biparametric MRI Radiomics Signature
Source: Contrast Media Mol Imaging. 2021 Dec 23;2021:7830909. doi: 10.1155/2021/7830909 (PMC8718299; doi:10.1155/2021/7830909)
Supplement: Supplementary Materials — The supplementary file contains the supplementary information about the original data for radiomics (Supplementary Materials and Supplement Tables 1–6). [file 7830909.f1.zip › 7830909.f1/Original data (1).pdf]

Indicator Test
AUC 0.885856
Precision 0.833333
Recall 0.967742
F-Score 0.895522
Lift 1.1828
2.1
Replace abnormal values by median;
Standardization.
2.2
M SUM 0 0 1 1
Data 142 41 101
g Training 98 28 70
g Testing 44 13 31
2.3 804， 8
Step 1:   Lasso: cross validation， 26
[1] "MinIntensity"
[2] "histogramEntropy"
[3] "Correlation_AllDirection_offset1_SD"
[4] "GLCMEntropy_AllDirection_offset1"
[5] "GLCMEntropy_AllDirection_offset4"
[6] "GLCMEntropy_AllDirection_offset7"
[7] "GLCMEntropy_angle0_offset1"
[8] "GLCMEntropy_angle0_offset4"
[9] "GLCMEntropy_angle0_offset7"
[10] "GLCMEntropy_angle135_offset1"
[11] "GLCMEntropy_angle135_offset4"
[12] "GLCMEntropy_angle135_offset7"
[13] "GLCMEntropy_angle45_offset1"
[14] "GLCMEntropy_angle45_offset4"
[15] "GLCMEntropy_angle90_offset1"
[16] "GLCMEntropy_angle90_offset4"
[17] "GLCMEntropy_angle90_offset7"
[18] "HaraEntroy"
[19] "sumAverage"
[20] "sumEntropy"
[21] "ShortRunHighGreyLevelEmphasis_AllDirection_offset1"
[22] "ShortRunHighGreyLevelEmphasis_angle0_offset1"
[23] "ShortRunHighGreyLevelEmphasis_angle0_offset7"
[24] "ShortRunHighGreyLevelEmphasis_angle45_offset1"
[25] "HighGreyLevelRunEmphasis_AllDirection_offset4_SD"
[26] "Elongation"
Step 2: spearman. 8
1

[1] "MinIntensity"
[2] "histogramEntropy"
[3] "Correlation_AllDirection_offset1_SD"
[4] "GLCMEntropy_AllDirection_offset1"
[5] "GLCMEntropy_angle0_offset7"
[6] "sumAverage"
[7] "HighGreyLevelRunEmphasis_AllDirection_offset4_SD"
[8] "Elongation"
2.4 LogisticRegression, 8
2.5：
Training Data's ROC Graph
Testing Data's ROC Graph
TestingPredictionGraph
CorrelationGraph
LassoCoefficientGraph
LassoErrorGraph
3.Log
Step 1: Load Data, the numbers of data total: 142; the numbers of feature total: 402.
Step 1: Load Data, the numbers of data total: 142; the numbers of feature total: 402.
Step 2: abnormal value processing. the selected method: Replace abnormal values by median.the result:
3
Step 2: abnormal value processing. the selected method: Replace abnormal values by median.the result:
contain abnormal value, their index and new value is: (2,46) ,8.77e+06; (3,46) ,8.77e+06; (4,46) ,8.77e+06; (5,46) ,8.77e+06; (6,46) ,8.77e+06; (7,46) ,8.77e+06; (10,46) ,8.77e+06; (11,46) ,8.77e+06; (13,46) ,8.77e+06; (14,46) ,8.77e+06; (22,46) ,8.77e+06; (24,46) ,8.77e+06; (28,46) ,8.77e+06; (33,46) ,8.77e+06; (36,46) ,8.77e+06; (37,46) ,8.77e+06; (38,46) ,8.77e+06; (39,46) ,8.77e+06; (49,46) ,8.77e+06; (64,46) ,8.77e+06; (69,46) ,8.77e+06; (86,46) ,8.77e+06; (119,46) ,8.77e+06; (132,46) ,8.77e+06; (138,46) ,8.77e+06; (143,46) ,8.77e+06; (2,47) ,4.6e+12; (3,47) ,4.6e+12; (4,47) ,4.6e+12; (5,47) ,4.6e+12; (6,47) ,4.6e+12; (7,47) ,4.6e+12; (10,47) ,4.6e+12; (11,47) ,4.6e+12; (13,47) ,4.6e+12; (14,47) ,4.6e+12; (22,47) ,4.6e+12; (24,47) ,4.6e+12; (28,47) ,4.6e+12; (33,47) ,4.6e+12; (36,47) ,4.6e+12; (37,47) ,4.6e+12; (38,47) ,4.6e+12; (39,47) ,4.6e+12; (49,47) ,4.6e+12; (64,47) ,4.6e+12; (69,47) ,4.6e+12; (86,47) ,4.6e+12; (119,47) ,4.6e+12; (132,47) ,4.6e+12; (138,47) ,4.6e+12; (143,47) ,4.6e+12; (2,48) ,8.03e+06; (3,48) ,8.03e+06; (4,48) ,8.03e+06;
(5,48) ,8.03e+06; (6,48) ,8.03e+06; (7,48) ,8.03e+06; (8,48) ,8.03e+06; (9,48) ,8.03e+06; (10,48) ,8.03e+06; (11,48) ,8.03e+06; (12,48) ,8.03e+06; (13,48) ,8.03e+06; (14,48) ,8.03e+06; (15,48) ,8.03e+06; (16,48) ,8.03e+06; (19,48) ,8.03e+06; (20,48) ,8.03e+06; (21,48) ,8.03e+06; (22,48) ,8.03e+06; (23,48) ,8.03e+06; (24,48) ,8.03e+06; (26,48) ,8.03e+06; (27,48) ,8.03e+06; (28,48) ,8.03e+06; (29,48) ,8.03e+06; (30,48) ,8.03e+06; (31,48) ,8.03e+06; (32,48) ,8.03e+06; (33,48) ,8.03e+06; (34,48) ,8.03e+06; (35,48) ,8.03e+06; (36,48) ,8.03e+06; (37,48) ,8.03e+06; (38,48) ,8.03e+06; (39,48) ,8.03e+06; (41,48) ,8.03e+06; (42,48) ,8.03e+06; (44,48) ,8.03e+06; (46,48) ,8.03e+06; (48,48) ,8.03e+06; (49,48) ,8.03e+06; (50,48) ,8.03e+06; (51,48) ,8.03e+06; (52,48)

,8.03e+06; (54,48) ,8.03e+06; (57,48) ,8.03e+06; (63,48) ,8.03e+06; (64,48) ,8.03e+06; (67,48)
,8.03e+06; (69,48) ,8.03e+06; (70,48) ,8.03e+06; (73,48) ,8.03e+06; (75,48) ,8.03e+06; (78,48)
,8.03e+06; (79,48) ,8.03e+06; (84,48) ,8.03e+06; (85,48) ,8.03e+06; (86,48) ,8.03e+06; (87,48)
,8.03e+06; (97,48) ,8.03e+06; (107,48) ,8.03e+06; (108,48) ,8.03e+06; (112,48) ,8.03e+06; (114,48)
,8.03e+06; (115,48) ,8.03e+06; (119,48) ,8.03e+06; (122,48) ,8.03e+06; (132,48) ,8.03e+06; (133,48)
,8.03e+06; (135,48) ,8.03e+06; (136,48) ,8.03e+06; (138,48) ,8.03e+06; (142,48) ,8.03e+06; (143,48)
,8.03e+06; (2,49) ,2.91e+12; (3,49) ,2.91e+12; (4,49) ,2.91e+12; (5,49) ,2.91e+12; (6,49) ,2.91e+12;
(7,49) ,2.91e+12; (8,49) ,2.91e+12; (9,49) ,2.91e+12; (10,49) ,2.91e+12; (11,49) ,2.91e+12; (12,49)
,2.91e+12; (13,49) ,2.91e+12; (14,49) ,2.91e+12; (15,49) ,2.91e+12; (16,49) ,2.91e+12; (19,49)
,2.91e+12; (20,49) ,2.91e+12; (21,49) ,2.91e+12; (22,49) ,2.91e+12; (23,49) ,2.91e+12; (24,49)
,2.91e+12; (26,49) ,2.91e+12; (27,49) ,2.91e+12; (28,49) ,2.91e+12; (29,49) ,2.91e+12; (30,49)
,2.91e+12; (31,49) ,2.91e+12; (32,49) ,2.91e+12; (33,49) ,2.91e+12; (34,49) ,2.91e+12; (35,49)
,2.91e+12; (36,49) ,2.91e+12; (37,49) ,2.91e+12; (38,49) ,2.91e+12; (39,49) ,2.91e+12; (41,49)
,2.91e+12; (42,49) ,2.91e+12; (44,49) ,2.91e+12; (46,49) ,2.91e+12; (48,49) ,2.91e+12; (49,49)
,2.91e+12; (50,49) ,2.91e+12; (51,49) ,2.91e+12; (52,49) ,2.91e+12; (54,49) ,2.91e+12; (57,49)
,2.91e+12; (63,49) ,2.91e+12; (64,49) ,2.91e+12; (67,49) ,2.91e+12; (69,49) ,2.91e+12; (70,49)
,2.91e+12; (73,49) ,2.91e+12; (75,49) ,2.91e+12; (78,49) ,2.91e+12; (79,49) ,2.91e+12; (84,49)
,2.91e+12; (85,49) ,2.91e+12; (86,49) ,2.91e+12; (87,49) ,2.91e+12; (97,49) ,2.91e+12; (107,49)
,2.91e+12; (108,49) ,2.91e+12; (112,49) ,2.91e+12; (114,49) ,2.91e+12; (115,49) ,2.91e+12; (119,49)
,2.91e+12; (122,49) ,2.91e+12; (132,49) ,2.91e+12; (133,49) ,2.91e+12; (135,49) ,2.91e+12; (136,49)
,2.91e+12; (138,49) ,2.91e+12; (142,49) ,2.91e+12; (143,49) ,2.91e+12; (2,51) ,8.57e+06; (3,51)
,8.57e+06; (4,51) ,8.57e+06; (5,51) ,8.57e+06; (13,51) ,8.57e+06; (28,51) ,8.57e+06; (2,52)
,7.22e+06; (3,52) ,7.22e+06; (4,52) ,7.22e+06; (5,52) ,7.22e+06; (6,52) ,7.22e+06; (8,52) ,7.22e+06;
(10,52) ,7.22e+06; (13,52) ,7.22e+06; (14,52) ,7.22e+06; (19,52) ,7.22e+06; (22,52) ,7.22e+06;
(24,52) ,7.22e+06; (28,52) ,7.22e+06; (30,52) ,7.22e+06; (33,52) ,7.22e+06; (35,52) ,7.22e+06;
(36,52) ,7.22e+06; (37,52) ,7.22e+06; (38,52) ,7.22e+06; (39,52) ,7.22e+06; (49,52) ,7.22e+06;
(52,52) ,7.22e+06; (64,52) ,7.22e+06; (69,52) ,7.22e+06; (86,52) ,7.22e+06; (119,52) ,7.22e+06;
(132,52) ,7.22e+06; (138,52) ,7.22e+06; (142,52) ,7.22e+06; (143,52) ,7.22e+06; (2,54) ,6.6e+06;
(3,54) ,6.6e+06; (4,54) ,6.6e+06; (5,54) ,6.6e+06; (7,54) ,6.6e+06; (10,54) ,6.6e+06; (11,54)
,6.6e+06; (13,54) ,6.6e+06; (28,54) ,6.6e+06; (33,54) ,6.6e+06; (36,54) ,6.6e+06; (37,54) ,6.6e+06;
(38,54) ,6.6e+06; (69,54) ,6.6e+06; (86,54) ,6.6e+06; (138,54) ,6.6e+06; (143,54) ,6.6e+06; (2,55)
,4.47e+06; (3,55) ,4.47e+06; (4,55) ,4.47e+06; (5,55) ,4.47e+06; (6,55) ,4.47e+06; (7,55) ,4.47e+06;
(9,55) ,4.47e+06; (10,55) ,4.47e+06; (11,55) ,4.47e+06; (12,55) ,4.47e+06; (13,55) ,4.47e+06; (14,55)
,4.47e+06; (15,55) ,4.47e+06; (16,55) ,4.47e+06; (19,55) ,4.47e+06; (21,55) ,4.47e+06; (22,55)
,4.47e+06; (23,55) ,4.47e+06; (24,55) ,4.47e+06; (26,55) ,4.47e+06; (28,55) ,4.47e+06; (29,55)

,4.47e+06; (30,55) ,4.47e+06; (31,55) ,4.47e+06; (32,55) ,4.47e+06; (33,55) ,4.47e+06; (35,55)
,4.47e+06; (36,55) ,4.47e+06; (37,55) ,4.47e+06; (38,55) ,4.47e+06; (39,55) ,4.47e+06; (41,55)
,4.47e+06; (42,55) ,4.47e+06; (46,55) ,4.47e+06; (49,55) ,4.47e+06; (50,55) ,4.47e+06; (52,55)
,4.47e+06; (54,55) ,4.47e+06; (57,55) ,4.47e+06; (64,55) ,4.47e+06; (67,55) ,4.47e+06; (69,55)
,4.47e+06; (70,55) ,4.47e+06; (75,55) ,4.47e+06; (86,55) ,4.47e+06; (97,55) ,4.47e+06; (107,55)
,4.47e+06; (108,55) ,4.47e+06; (114,55) ,4.47e+06; (115,55) ,4.47e+06; (119,55) ,4.47e+06; (133,55)
,4.47e+06; (138,55) ,4.47e+06; (142,55) ,4.47e+06; (143,55) ,4.47e+06; (2,57) ,6.58e+06; (3,57)
,6.58e+06; (4,57) ,6.58e+06; (5,57) ,6.58e+06; (6,57) ,6.58e+06; (7,57) ,6.58e+06; (14,57) ,6.58e+06;
(22,57) ,6.58e+06; (24,57) ,6.58e+06; (28,57) ,6.58e+06; (33,57) ,6.58e+06; (36,57) ,6.58e+06;
(38,57) ,6.58e+06; (39,57) ,6.58e+06; (49,57) ,6.58e+06; (64,57) ,6.58e+06; (86,57) ,6.58e+06;
(119,57) ,6.58e+06; (132,57) ,6.58e+06; (138,57) ,6.58e+06; (2,58) ,6.56e+06; (3,58) ,6.56e+06;
(4,58) ,6.56e+06; (5,58) ,6.56e+06; (6,58) ,6.56e+06; (7,58) ,6.56e+06; (8,58) ,6.56e+06; (9,58)
,6.56e+06; (10,58) ,6.56e+06; (12,58) ,6.56e+06; (13,58) ,6.56e+06; (14,58) ,6.56e+06; (15,58)

,6.56e+06; (10,58) ,6.56e+06; (12,58) ,6.56e+06; (13,58) ,6.56e+06; (14,58) ,6.56e+06; (15,58)
,6.56e+06; (16,58) ,6.56e+06; (20,58) ,6.56e+06; (21,58) ,6.56e+06; (22,58) ,6.56e+06; (24,58)
,6.56e+06; (26,58) ,6.56e+06; (27,58) ,6.56e+06; (28,58) ,6.56e+06; (29,58) ,6.56e+06; (30,58)
,6.56e+06; (32,58) ,6.56e+06; (33,58) ,6.56e+06; (34,58) ,6.56e+06; (35,58) ,6.56e+06; (36,58)
,6.56e+06; (37,58) ,6.56e+06; (38,58) ,6.56e+06; (39,58) ,6.56e+06; (42,58) ,6.56e+06; (44,58)
,6.56e+06; (46,58) ,6.56e+06; (48,58) ,6.56e+06; (49,58) ,6.56e+06; (50,58) ,6.56e+06; (51,58)
,6.56e+06; (52,58) ,6.56e+06; (54,58) ,6.56e+06; (63,58) ,6.56e+06; (64,58) ,6.56e+06; (67,58)
,6.56e+06; (69,58) ,6.56e+06; (70,58) ,6.56e+06; (73,58) ,6.56e+06; (75,58) ,6.56e+06; (78,58)
,6.56e+06; (79,58) ,6.56e+06; (84,58) ,6.56e+06; (85,58) ,6.56e+06; (86,58) ,6.56e+06; (87,58)
,6.56e+06; (107,58) ,6.56e+06; (108,58) ,6.56e+06; (112,58) ,6.56e+06; (114,58) ,6.56e+06; (119,58)
,6.56e+06; (122,58) ,6.56e+06; (132,58) ,6.56e+06; (135,58) ,6.56e+06; (136,58) ,6.56e+06; (138,58)
,6.56e+06; (142,58) ,6.56e+06; (143,58) ,6.56e+06; (2,60) ,7.57e+06; (3,60) ,7.57e+06; (4,60)
,7.57e+06; (5,60) ,7.57e+06; (6,60) ,7.57e+06; (36,60) ,7.57e+06; (138,60) ,7.57e+06; (2,61)
,5.68e+06; (3,61) ,5.68e+06; (4,61) ,5.68e+06; (5,61) ,5.68e+06; (6,61) ,5.68e+06; (7,61) ,5.68e+06;
(11,61) ,5.68e+06; (12,61) ,5.68e+06; (13,61) ,5.68e+06; (14,61) ,5.68e+06; (15,61) ,5.68e+06;
(21,61) ,5.68e+06; (24,61) ,5.68e+06; (26,61) ,5.68e+06; (28,61) ,5.68e+06; (29,61) ,5.68e+06;
(32,61) ,5.68e+06; (33,61) ,5.68e+06; (35,61) ,5.68e+06; (36,61) ,5.68e+06; (37,61) ,5.68e+06;
(38,61) ,5.68e+06; (42,61) ,5.68e+06; (46,61) ,5.68e+06; (49,61) ,5.68e+06; (50,61) ,5.68e+06;
(52,61) ,5.68e+06; (54,61) ,5.68e+06; (69,61) ,5.68e+06; (70,61) ,5.68e+06; (84,61) ,5.68e+06;
(86,61) ,5.68e+06; (119,61) ,5.68e+06; (138,61) ,5.68e+06; (142,61) ,5.68e+06; (143,61) ,5.68e+06;
(2,64) ,29437.2; (3,64) ,29437.2; (4,64) ,29437.2; (5,64) ,29437.2; (6,64) ,29437.2; (7,64) ,29437.2;
(10,64) ,29437.2; (11,64) ,29437.2; (13,64) ,29437.2; (14,64) ,29437.2; (22,64) ,29437.2; (24,64)
,29437.2; (28,64) ,29437.2; (33,64) ,29437.2; (36,64) ,29437.2; (37,64) ,29437.2; (38,64) ,29437.2;

(39,64) ,29437.2; (49,64) ,29437.2; (64,64) ,29437.2; (69,64) ,29437.2; (86,64) ,29437.2; (119,64) ,29437.2; (132,64) ,29437.2; (138,64) ,29437.2; (143,64) ,29437.2; (2,65) ,1.43e+08; (3,65) ,1.43e+08; (4,65) ,1.43e+08; (5,65) ,1.43e+08; (6,65) ,1.43e+08; (7,65) ,1.43e+08; (10,65) ,1.43e+08;

(11,65) ,1.43e+08; (13,65) ,1.43e+08; (14,65) ,1.43e+08; (22,65) ,1.43e+08; (24,65) ,1.43e+08; (28,65) ,1.43e+08; (33,65) ,1.43e+08; (36,65) ,1.43e+08; (37,65) ,1.43e+08; (38,65) ,1.43e+08; (39,65) ,1.43e+08; (49,65) ,1.43e+08; (64,65) ,1.43e+08; (69,65) ,1.43e+08; (86,65) ,1.43e+08; (119,65) ,1.43e+08; (132,65) ,1.43e+08; (138,65) ,1.43e+08; (143,65) ,1.43e+08; (2,66) ,34180.6; (3,66) ,34180.6; (4,66) ,34180.6; (5,66) ,34180.6; (6,66) ,34180.6; (7,66) ,34180.6; (8,66) ,34180.6;

(9,66) ,34180.6; (10,66) ,34180.6; (11,66) ,34180.6; (12,66) ,34180.6; (13,66) ,34180.6; (14,66) ,34180.6; (15,66) ,34180.6; (16,66) ,34180.6; (19,66) ,34180.6; (20,66) ,34180.6; (21,66) ,34180.6; (22,66) ,34180.6; (23,66) ,34180.6; (24,66) ,34180.6; (26,66) ,34180.6; (27,66) ,34180.6; (28,66) ,34180.6; (29,66) ,34180.6; (30,66) ,34180.6; (31,66) ,34180.6; (32,66) ,34180.6; (33,66) ,34180.6; (34,66) ,34180.6; (35,66) ,34180.6; (36,66) ,34180.6; (37,66) ,34180.6; (38,66) ,34180.6; (39,66) ,34180.6; (41,66) ,34180.6; (42,66) ,34180.6; (44,66) ,34180.6; (46,66) ,34180.6; (48,66) ,34180.6; (49,66) ,34180.6; (50,66) ,34180.6; (51,66) ,34180.6; (52,66) ,34180.6; (54,66) ,34180.6; (57,66) ,34180.6; (63,66) ,34180.6; (64,66) ,34180.6; (67,66) ,34180.6; (69,66) ,34180.6; (70,66) ,34180.6; (73,66) ,34180.6; (75,66) ,34180.6; (78,66) ,34180.6; (79,66) ,34180.6; (84,66) ,34180.6; (85,66) ,34180.6; (86,66) ,34180.6; (87,66) ,34180.6; (97,66) ,34180.6; (107,66) ,34180.6; (108,66) ,34180.6;

(112,66) ,34180.6; (114,66) ,34180.6; (115,66) ,34180.6; (119,66) ,34180.6; (122,66) ,34180.6; (132,66) ,34180.6; (133,66) ,34180.6; (135,66) ,34180.6; (136,66) ,34180.6; (138,66) ,34180.6; (142,66) ,34180.6; (143,66) ,34180.6; (2,67) ,9.69e+07; (3,67) ,9.69e+07; (4,67) ,9.69e+07; (5,67) ,9.69e+07; (6,67) ,9.69e+07; (7,67) ,9.69e+07; (8,67) ,9.69e+07; (9,67) ,9.69e+07; (10,67) ,9.69e+07;

(11,67) ,9.69e+07; (12,67) ,9.69e+07; (13,67) ,9.69e+07; (14,67) ,9.69e+07; (15,67) ,9.69e+07; (16,67) ,9.69e+07; (19,67) ,9.69e+07; (20,67) ,9.69e+07; (21,67) ,9.69e+07; (22,67) ,9.69e+07; (23,67) ,9.69e+07; (24,67) ,9.69e+07; (26,67) ,9.69e+07; (27,67) ,9.69e+07; (28,67) ,9.69e+07; (29,67) ,9.69e+07; (30,67) ,9.69e+07; (31,67) ,9.69e+07; (32,67) ,9.69e+07; (33,67) ,9.69e+07; (34,67) ,9.69e+07; (35,67) ,9.69e+07; (36,67) ,9.69e+07; (37,67) ,9.69e+07; (38,67) ,9.69e+07; (39,67) ,9.69e+07; (41,67) ,9.69e+07; (42,67) ,9.69e+07; (44,67) ,9.69e+07; (46,67) ,9.69e+07; (48,67) ,9.69e+07; (49,67) ,9.69e+07; (50,67) ,9.69e+07; (51,67) ,9.69e+07; (52,67) ,9.69e+07; (54,67) ,9.69e+07; (57,67) ,9.69e+07; (63,67) ,9.69e+07; (64,67) ,9.69e+07; (67,67) ,9.69e+07; (69,67) ,9.69e+07; (70,67) ,9.69e+07; (73,67) ,9.69e+07; (75,67) ,9.69e+07; (78,67) ,9.69e+07; (79,67) ,9.69e+07; (84,67) ,9.69e+07; (85,67) ,9.69e+07; (86,67) ,9.69e+07; (87,67) ,9.69e+07; (97,67) ,9.69e+07; (107,67) ,9.69e+07; (108,67) ,9.69e+07; (112,67) ,9.69e+07; (114,67) ,9.69e+07;

(115,67) ,9.69e+07; (119,67) ,9.69e+07; (122,67) ,9.69e+07; (132,67) ,9.69e+07; (133,67) ,9.69e+07;

(135,67) ,9.69e+07; (136,67) ,9.69e+07; (138,67) ,9.69e+07; (142,67) ,9.69e+07; (143,67) ,9.69e+07;

(2,69) ,32044.4; (3,69) ,32044.4; (4,69) ,32044.4; (5,69) ,32044.4; (13,69) ,32044.4; (28,69) ,32044.4; (2,70) ,30224.6; (3,70) ,30224.6; (4,70) ,30224.6; (5,70) ,30224.6; (6,70) ,30224.6;

(8,70)
,30224.6; (10,70) ,30224.6; (13,70) ,30224.6; (14,70) ,30224.6; (19,70) ,30224.6; (22,70) ,30224.6; (24,70) ,30224.6; (28,70) ,30224.6; (30,70) ,30224.6; (33,70) ,30224.6; (35,70) ,30224.6; (36,70) ,30224.6; (37,70) ,30224.6; (38,70) ,30224.6; (39,70) ,30224.6; (49,70) ,30224.6; (52,70) ,30224.6; (64,70) ,30224.6; (69,70) ,30224.6; (86,70) ,30224.6; (119,70) ,30224.6; (132,70) ,30224.6; (138,70)
,30224.6; (142,70) ,30224.6; (143,70) ,30224.6; (2,72) ,25195.8; (3,72) ,25195.8; (4,72) ,25195.8; (5,72) ,25195.8; (7,72) ,25195.8; (10,72) ,25195.8; (11,72) ,25195.8; (13,72) ,25195.8; (28,72) ,25195.8; (33,72) ,25195.8; (36,72) ,25195.8; (37,72) ,25195.8; (38,72) ,25195.8; (69,72) ,25195.8; (86,72) ,25195.8; (138,72) ,25195.8; (143,72) ,25195.8; (2,73) ,22036.5; (3,73) ,22036.5; (4,73)

(86,72) ,25195.8; (138,72) ,25195.8; (143,72) ,25195.8; (2,73) ,22036.5; (3,73) ,22036.5; (4,73)
,22036.5; (5,73) ,22036.5; (6,73) ,22036.5; (7,73) ,22036.5; (9,73) ,22036.5; (10,73) ,22036.5; (11,73) ,22036.5; (12,73) ,22036.5; (13,73) ,22036.5; (14,73) ,22036.5; (15,73) ,22036.5; (16,73) ,22036.5; (19,73) ,22036.5; (21,73) ,22036.5; (22,73) ,22036.5; (23,73) ,22036.5; (24,73) ,22036.5; (26,73) ,22036.5; (28,73) ,22036.5; (29,73) ,22036.5; (30,73) ,22036.5; (31,73) ,22036.5; (32,73) ,22036.5; (33,73) ,22036.5; (35,73) ,22036.5; (36,73) ,22036.5; (37,73) ,22036.5; (38,73) ,22036.5; (39,73) ,22036.5; (41,73) ,22036.5; (42,73) ,22036.5; (46,73) ,22036.5; (49,73) ,22036.5; (50,73) ,22036.5; (52,73) ,22036.5; (54,73) ,22036.5; (57,73) ,22036.5; (64,73) ,22036.5; (67,73) ,22036.5; (69,73) ,22036.5; (70,73) ,22036.5; (75,73) ,22036.5; (86,73) ,22036.5; (97,73) ,22036.5; (107,73) ,22036.5; (108,73) ,22036.5; (114,73) ,22036.5; (115,73) ,22036.5; (119,73) ,22036.5; (133,73) ,22036.5; (138,73) ,22036.5; (142,73) ,22036.5; (143,73) ,22036.5; (2,75) ,23547.7; (3,75) ,23547.7;
(4,75) ,23547.7; (5,75) ,23547.7; (6,75) ,23547.7; (7,75) ,23547.7; (14,75) ,23547.7; (22,75) ,23547.7; (24,75) ,23547.7; (28,75) ,23547.7; (33,75) ,23547.7; (36,75) ,23547.7; (38,75) ,23547.7; (39,75) ,23547.7; (49,75) ,23547.7; (64,75) ,23547.7; (86,75) ,23547.7; (119,75) ,23547.7; (132,75)
,23547.7; (138,75) ,23547.7; (2,76) ,28054.2; (3,76) ,28054.2; (4,76) ,28054.2; (5,76) ,28054.2; (6,76) ,28054.2; (7,76) ,28054.2; (8,76) ,28054.2; (9,76) ,28054.2; (10,76) ,28054.2; (12,76) ,28054.2; (13,76) ,28054.2; (14,76) ,28054.2; (15,76) ,28054.2; (16,76) ,28054.2; (20,76) ,28054.2; (21,76) ,28054.2; (22,76) ,28054.2; (24,76) ,28054.2; (26,76) ,28054.2; (27,76) ,28054.2; (28,76) ,28054.2; (29,76) ,28054.2; (30,76) ,28054.2; (32,76) ,28054.2; (33,76) ,28054.2; (34,76) ,28054.2; (35,76) ,28054.2; (36,76) ,28054.2; (37,76) ,28054.2; (38,76) ,28054.2; (39,76) ,28054.2; (42,76) ,28054.2; (44,76) ,28054.2; (46,76) ,28054.2; (48,76) ,28054.2; (49,76) ,28054.2; (50,76) ,28054.2; (51,76) ,28054.2; (52,76) ,28054.2; (54,76) ,28054.2; (63,76) ,28054.2; (64,76) ,28054.2; (67,76) ,28054.2; (69,76) ,28054.2; (70,76) ,28054.2; (73,76) ,28054.2; (75,76) ,28054.2; (78,76) ,28054.2; (79,76) ,28054.2; (84,76) ,28054.2; (85,76) ,28054.2; (86,76) ,28054.2; (87,76) ,28054.2; (107,76) ,28054.2; (108,76) ,28054.2; (112,76) ,28054.2; (114,76) ,28054.2; (119,76) ,28054.2; (122,76) ,28054.2; (132,76) ,28054.2; (135,76) ,28054.2; (136,76) ,28054.2; (138,76) ,28054.2; (142,76) ,28054.2; (143,76) ,28054.2; (2,78) ,26500.2; (3,78) ,26500.2; (4,78) ,26500.2; (5,78) ,26500.2; (6,78) ,26500.2; (36,78) ,26500.2; (138,78) ,26500.2; (2,79) ,23613.7; (3,79) ,23613.7; (4,79) ,23613.7; (5,79) ,23613.7; (6,79) ,23613.7; (7,79) ,23613.7; (11,79) ,23613.7; (12,79) ,23613.7; (13,79) ,23613.7; (14,79) ,23613.7; (15,79) ,23613.7; (21,79) ,23613.7; (24,79) ,23613.7; (26,79) ,23613.7; (28,79) ,23613.7; (29,79) ,23613.7; (32,79) ,23613.7; (33,79) ,23613.7; (35,79) ,23613.7;

(36,79) ,23613.7; (37,79) ,23613.7; (38,79) ,23613.7; (42,79) ,23613.7; (46,79) ,23613.7; (49,79) ,23613.7; (50,79) ,23613.7; (52,79) ,23613.7; (54,79) ,23613.7; (69,79) ,23613.7; (70,79) ,23613.7; (84,79) ,23613.7; (86,79) ,23613.7; (119,79) ,23613.7; (138,79) ,23613.7; (142,79) ,23613.7; (143,79)
,23613.7; (2,82) ,0.000106345; (3,82) ,0.000106345; (4,82) ,0.000106345; (5,82) ,0.000106345; (6,82)
,0.000106345; (7,82) ,0.000106345; (10,82) ,0.000106345; (11,82) ,0.000106345; (13,82) ,0.000106345;
(14,82) ,0.000106345; (22,82) ,0.000106345; (24,82) ,0.000106345; (28,82) ,0.000106345; (33,82)
,0.000106345; (36,82) ,0.000106345; (37,82) ,0.000106345; (38,82) ,0.000106345; (39,82) ,0.000106345;
(49,82) ,0.000106345; (64,82) ,0.000106345; (69,82) ,0.000106345; (86,82) ,0.000106345; (119,82)
,0.000106345; (132,82) ,0.000106345; (138,82) ,0.000106345; (143,82) ,0.000106345; (2,83) ,2.67e-08;
(3,83) ,2.67e-08; (4,83) ,2.67e-08; (5,83) ,2.67e-08; (6,83) ,2.67e-08; (7,83) ,2.67e-08; (10,83) ,2.67e-08; (11,83) ,2.67e-08; (13,83) ,2.67e-08; (14,83) ,2.67e-08; (22,83) ,2.67e-08; (24,83) ,2.67e-08; (28,83) ,2.67e-08; (33,83) ,2.67e-08; (36,83) ,2.67e-08; (37,83) ,2.67e-08; (38,83) ,2.67e-08; (39,83) ,2.67e-08; (49,83) ,2.67e-08; (64,83) ,2.67e-08; (69,83) ,2.67e-08; (86,83) ,2.67e-08; (119,83) ,2.67e-08; (132,83) ,2.67e-08; (138,83) ,2.67e-08; (143,83) ,2.67e-08; (2,84) ,3.02e-05; (3,84) ,3.02e-05; (4,84) ,3.02e-05; (5,84) ,3.02e-05; (6,84) ,3.02e-05; (7,84) ,3.02e-05; (8,84) ,3.02e-05; (9,84) ,3.02e-05; (10,84) ,3.02e-05; (11,84) ,3.02e-05; (12,84) ,3.02e-05; (13,84) ,3.02e-05; (14,84) ,3.02e-05; (15,84) ,3.02e-05; (16,84) ,3.02e-05; (19,84) ,3.02e-05; (20,84) ,3.02e-05; (21,84) ,3.02e-05; (22,84) ,3.02e-05; (23,84) ,3.02e-05; (24,84) ,3.02e-05; (26,84) ,3.02e-05; (27,84) ,3.02e-05; (28,84) ,3.02e-05; (29,84) ,3.02e-05; (30,84) ,3.02e-05; (31,84) ,3.02e-05; (32,84) ,3.02e-05; (33,84) ,3.02e-05; (34,84) ,3.02e-05; (35,84) ,3.02e-05; (36,84) ,3.02e-05; (37,84) ,3.02e-05; (38,84) ,3.02e-05; (39,84) ,3.02e-05; (41,84) ,3.02e-05; (42,84) ,3.02e-05; (44,84) ,3.02e-05; (46,84) ,3.02e-05; (48,84) ,3.02e-05; (49,84) ,3.02e-05; (50,84) ,3.02e-05; (51,84) ,3.02e-05; (52,84) ,3.02e-05; (54,84) ,3.02e-05; (57,84) ,3.02e-05; (63,84) ,3.02e-05; (64,84) ,3.02e-05; (67,84) ,3.02e-05; (69,84) ,3.02e-05; (70,84) ,3.02e-05; (73,84) ,3.02e-05; (75,84) ,3.02e-05; (78,84) ,3.02e-05; (79,84) ,3.02e-05; (84,84) ,3.02e-05; (85,84) ,3.02e-05; (86,84) ,3.02e-05; (87,84) ,3.02e-05; (97,84) ,3.02e-05; (107,84) ,3.02e-05; (108,84) ,3.02e-05; (112,84) ,3.02e-05; (114,84) ,3.02e-05; (115,84) ,3.02e-05; (119,84) ,3.02e-05; (122,84) ,3.02e-05; (132,84) ,3.02e-05; (133,84) ,3.02e-05; (135,84) ,3.02e-05; (136,84) ,3.02e-05; (138,84) ,3.02e-05; (142,84) ,3.02e-05; (143,84) ,3.02e-05; (2,85) ,2.2e-08; (3,85) ,2.2e-08; (4,85) ,2.2e-08; (5,85) ,2.2e-08; (6,85) ,2.2e-08; (7,85) ,2.2e-08; (8,85) ,2.2e-08; (9,85) ,2.2e-08; (10,85) ,2.2e-08; (11,85) ,2.2e-08; (12,85) ,2.2e-08; (13,85) ,2.2e-08; (14,85) ,2.2e-08; (15,85) ,2.2e-08; (16,85) ,2.2e-08; (19,85) ,2.2e-08; (20,85) ,2.2e-08; (21,85) ,2.2e-08; (22,85) ,2.2e-08; (23,85) ,2.2e-08; (24,85) ,2.2e-08; (26,85) ,2.2e-08; (27,85) ,2.2e-08; (28,85) ,2.2e-08; (29,85) ,2.2e-08; (30,85) ,2.2e-08; (31,85) ,2.2e-08; (32,85) ,2.2e-08; (33,85) ,2.2e-08; (34,85) ,2.2e-08; (35,85) ,2.2e-08; (36,85) ,2.2e-08; (37,85) ,2.2e-08; (38,85) ,2.2e-08; (39,85) ,2.2e-08; (41,85) ,2.2e-08; (42,85) ,2.2e-08; (44,85) ,2.2e-08; (46,85) ,2.2e-08; (48,85) ,2.2e-08; (49,85) ,2.2e-08; (50,85) ,2.2e-08; (51,85) ,2.2e-08; (52,85) ,2.2e-08; (54,85) ,2.2e-08; (57,85) ,2.2e-08; (63,85) ,2.2e-08;

6

,2.2e-08; (51,85) ,2.2e-08; (52,85) ,2.2e-08; (54,85) ,2.2e-08; (57,85) ,2.2e-08; (63,85) ,2.2e-08; (64,85) ,2.2e-08; (67,85) ,2.2e-08; (69,85) ,2.2e-08; (70,85) ,2.2e-08; (73,85) ,2.2e-08; (75,85) ,2.2e-08; (78,85) ,2.2e-08; (79,85) ,2.2e-08; (84,85) ,2.2e-08; (85,85) ,2.2e-08; (86,85) ,2.2e-08; (87,85) ,2.2e-08; (97,85) ,2.2e-08; (107,85) ,2.2e-08; (108,85) ,2.2e-08; (112,85) ,2.2e-08; (114,85)

,2.2e-08; (115,85) ,2.2e-08; (119,85) ,2.2e-08; (122,85) ,2.2e-08; (132,85) ,2.2e-08; (133,85) ,2.2e-08; (135,85) ,2.2e-08; (136,85) ,2.2e-08; (138,85) ,2.2e-08; (142,85) ,2.2e-08; (143,85) ,2.2e-08; (2,87) ,0.000200861; (3,87) ,0.000200861; (4,87) ,0.000200861; (5,87) ,0.000200861; (13,87)

,0.000200861; (28,87) ,0.000200861; (2,88) ,5.44e-05; (3,88) ,5.44e-05; (4,88) ,5.44e-05; (5,88) ,5.44e-05; (6,88) ,5.44e-05; (8,88) ,5.44e-05; (10,88) ,5.44e-05; (13,88) ,5.44e-05; (14,88) ,5.44e-05; (19,88) ,5.44e-05; (22,88) ,5.44e-05; (24,88) ,5.44e-05; (28,88) ,5.44e-05; (30,88) ,5.44e-05; (33,88) ,5.44e-05; (35,88) ,5.44e-05; (36,88) ,5.44e-05; (37,88) ,5.44e-05; (38,88) ,5.44e-05; (39,88) ,5.44e-05; (49,88) ,5.44e-05; (52,88) ,5.44e-05; (64,88) ,5.44e-05; (69,88) ,5.44e-05; (86,88) ,5.44e-05; (119,88) ,5.44e-05; (132,88) ,5.44e-05; (138,88) ,5.44e-05; (142,88) ,5.44e-05; (143,88) ,5.44e-05; (2,90) ,8.23e-06; (3,90) ,8.23e-06; (4,90) ,8.23e-06; (5,90) ,8.23e-06; (7,90) ,8.23e-06; (10,90) ,8.23e-06; (11,90) ,8.23e-06; (13,90) ,8.23e-06; (28,90) ,8.23e-06; (33,90) ,8.23e-06; (36,90) ,8.23e-06; (37,90) ,8.23e-06; (38,90) ,8.23e-06; (49,90) ,8.23e-06; (69,90) ,8.23e-06; (86,90) ,8.23e-06; (138,90) ,8.23e-06; (143,90) ,8.23e-06; (2,91) ,-7.76e-05; (3,91) ,-7.76e-05; (4,91) ,-7.76e-05; (5,91) ,-7.76e-05; (6,91) ,-7.76e-05; (7,91) ,-7.76e-05; (9,91) ,-7.76e-05; (10,91) ,-7.76e-05; (11,91) ,-7.76e-05; (12,91) ,-7.76e-05; (13,91) ,-7.76e-05; (14,91) ,-7.76e-05; (15,91) ,-7.76e-05; (16,91) ,-7.76e-05; (19,91) ,-7.76e-05; (21,91) ,-7.76e-05; (22,91) ,-7.76e-05; (23,91) ,-7.76e-05; (24,91) ,-7.76e-05; (26,91) ,-7.76e-05; (28,91) ,-7.76e-05; (29,91) ,-7.76e-05; (30,91) ,-7.76e-05; (31,91) ,-7.76e-05; (32,91) ,-7.76e-05; (33,91) ,-7.76e-05; (35,91) ,-7.76e-05; (36,91) ,-7.76e-05; (37,91) ,-7.76e-05; (38,91) ,-7.76e-05; (39,91) ,-7.76e-05; (41,91) ,-7.76e-05; (42,91) ,-7.76e-05; (46,91) ,-7.76e-05; (49,91) ,-7.76e-05; (50,91) ,-7.76e-05; (52,91) ,-7.76e-05; (54,91) ,-7.76e-05; (57,91) ,-7.76e-05; (64,91) ,-7.76e-05; (67,91) ,-7.76e-05; (69,91) ,-7.76e-05; (70,91) ,-7.76e-05; (75,91) ,-7.76e-05; (86,91) ,-7.76e-05; (97,91) ,-7.76e-05; (107,91) ,-7.76e-05; (108,91) ,-7.76e-05; (114,91) ,-7.76e-05; (115,91) ,-7.76e-05; (119,91) ,-7.76e-05; (133,91) ,-7.76e-05; (138,91) ,-7.76e-05; (142,91) ,-7.76e-05; (143,91) ,-7.76e-05; (2,93) ,5.93e-05; (3,93) ,5.93e-05; (4,93) ,5.93e-05; (5,93) ,5.93e-05; (6,93) ,5.93e-05; (7,93) ,5.93e-05; (14,93) ,5.93e-05; (22,93) ,5.93e-05; (24,93) ,5.93e-05; (28,93) ,5.93e-05; (33,93)

,5.93e-05; (36,93) ,5.93e-05; (38,93) ,5.93e-05; (39,93) ,5.93e-05; (49,93) ,5.93e-05; (64,93) ,5.93e-05; (86,93) ,5.93e-05; (119,93) ,5.93e-05; (132,93) ,5.93e-05; (138,93) ,5.93e-05; (2,94) ,-3.69e-05; (3,94) ,-3.69e-05; (4,94) ,-3.69e-05; (5,94) ,-3.69e-05; (6,94) ,-3.69e-05; (7,94) ,-3.69e-05; (8,94) ,-3.69e-05; (9,94) ,-3.69e-05; (10,94) ,-3.69e-05; (12,94) ,-3.69e-05; (13,94) ,-3.69e-05; (14,94) ,-3.69e-05; (15,94) ,-3.69e-05; (16,94) ,-3.69e-05; (20,94) ,-3.69e-05; (21,94) ,-3.69e-05; (22,94) ,-3.69e-05; (24,94) ,-3.69e-05; (26,94) ,-3.69e-05; (27,94) ,-3.69e-05; (28,94) ,-3.69e-05; (29,94) ,-3.69e-05; (30,94) ,-3.69e-05; (32,94) ,-3.69e-05; (33,94) ,-3.69e-05; (34,94) ,-3.69e-05; (35,94) ,-3.69e-05; (36,94) ,-3.69e-05; (37,94) ,-3.69e-05; (38,94) ,-3.69e-05; (39,94) ,-3.69e-05; (42,94) ,-3.69e-05; (44,94) ,-3.69e-05; (46,94) ,-3.69e-05; (48,94) ,-3.69e-05; (49,94) ,-3.69e-05; (50,94) ,-3.69e-05; (51,94) ,-3.69e-05; (52,94) ,-3.69e-05; (54,94) ,-3.69e-05; (63,94)

,-3.69e-05; (64,94) ,-3.69e-05; (67,94) ,-3.69e-05; (69,94) ,-3.69e-05; (70,94) ,-3.69e-05; (73,94)
,-3.69e-05; (75,94) ,-3.69e-05; (78,94) ,-3.69e-05; (79,94) ,-3.69e-05; (84,94) ,-3.69e-05; (85,94)
,-3.69e-05; (86,94) ,-3.69e-05; (87,94) ,-3.69e-05; (107,94) ,-3.69e-05; (108,94) ,-3.69e-05;
(112,94) ,-3.69e-05; (114,94) ,-3.69e-05; (119,94) ,-3.69e-05; (122,94) ,-3.69e-05; (132,94)
,-3.69e-05; (135,94) ,-3.69e-05; (136,94) ,-3.69e-05; (138,94) ,-3.69e-05; (142,94) ,-3.69e-05;
(143,94) ,-3.69e-05; (2,96) ,0.000100539; (3,96) ,0.000100539; (4,96) ,0.000100539; (5,96)
,0.000100539; (6,96) ,0.000100539; (36,96) ,0.000100539; (138,96) ,0.000100539;
(2,97) ,2.48e-05;
(3,97) ,2.48e-05; (4,97) ,2.48e-05; (5,97) ,2.48e-05; (6,97) ,2.48e-05; (7,97) ,2.48e-05; (11,97)
,2.48e-05; (12,97) ,2.48e-05; (13,97) ,2.48e-05; (14,97) ,2.48e-05; (15,97) ,2.48e-05; (21,97)
,2.48e-05; (24,97) ,2.48e-05; (26,97) ,2.48e-05; (28,97) ,2.48e-05; (29,97) ,2.48e-05; (32,97)
,2.48e-05; (33,97) ,2.48e-05; (35,97) ,2.48e-05; (36,97) ,2.48e-05; (37,97) ,2.48e-05; (38,97)
,2.48e-05; (42,97) ,2.48e-05; (46,97) ,2.48e-05; (49,97) ,2.48e-05; (50,97) ,2.48e-05; (52,97)
,2.48e-05; (54,97) ,2.48e-05; (69,97) ,2.48e-05; (70,97) ,2.48e-05; (84,97) ,2.48e-05; (86,97)
,2.48e-05; (119,97) ,2.48e-05; (138,97) ,2.48e-05; (142,97) ,2.48e-05; (143,97) ,2.48e-05; (2,100)
,0.00344544; (3,100) ,0.00344544; (4,100) ,0.00344544; (5,100) ,0.00344544;
(6,100) ,0.00344544;
(7,100) ,0.00344544; (10,100) ,0.00344544; (11,100) ,0.00344544; (13,100) ,0.00344544;
(14,100)
,0.00344544; (22,100) ,0.00344544; (24,100) ,0.00344544; (28,100) ,0.00344544;
(33,100) ,0.00344544;
(36,100) ,0.00344544; (37,100) ,0.00344544; (38,100) ,0.00344544; (39,100) ,0.00344544;
(49,100)
,0.00344544; (64,100) ,0.00344544; (69,100) ,0.00344544; (86,100) ,0.00344544;
(119,100) ,0.00344544;
(132,100) ,0.00344544; (138,100) ,0.00344544; (143,100) ,0.00344544; (2,101) ,7.78e-07; (3,101)
,7.78e-07; (4,101) ,7.78e-07; (5,101) ,7.78e-07; (6,101) ,7.78e-07; (7,101) ,7.78e-07; (10,101)
,7.78e-07; (11,101) ,7.78e-07; (13,101) ,7.78e-07; (14,101) ,7.78e-07; (22,101) ,7.78e-07; (24,101)
,7.78e-07; (28,101) ,7.78e-07; (33,101) ,7.78e-07; (36,101) ,7.78e-07; (37,101) ,7.78e-07; (38,101)
,7.78e-07; (39,101) ,7.78e-07; (49,101) ,7.78e-07; (64,101) ,7.78e-07; (69,101) ,7.78e-07; (86,101)
,7.78e-07; (119,101) ,7.78e-07; (132,101) ,7.78e-07; (138,101) ,7.78e-07; (143,101) ,7.78e-07;
(2,102) ,0.00088249; (3,102) ,0.00088249; (4,102) ,0.00088249; (5,102) ,0.00088249; (6,102)
,0.00088249; (7,102) ,0.00088249; (8,102) ,0.00088249; (9,102) ,0.00088249;
(10,102) ,0.00088249;
(11,102) ,0.00088249; (12,102) ,0.00088249; (13,102) ,0.00088249; (14,102) ,0.00088249;
(15,102)
7
(11,102) ,0.00088249; (12,102) ,0.00088249; (13,102) ,0.00088249; (14,102) ,0.00088249;
(15,102)
,0.00088249; (16,102) ,0.00088249; (19,102) ,0.00088249; (20,102) ,0.00088249;
(21,102) ,0.00088249;
(22,102) ,0.00088249; (23,102) ,0.00088249; (24,102) ,0.00088249; (26,102) ,0.00088249;
(27,102)
,0.00088249; (28,102) ,0.00088249; (29,102) ,0.00088249; (30,102) ,0.00088249;

(31,102) ,0.00088249;
(32,102) ,0.00088249; (33,102) ,0.00088249; (34,102) ,0.00088249; (35,102) ,0.00088249; (36,102)
,0.00088249; (37,102) ,0.00088249; (38,102) ,0.00088249; (39,102) ,0.00088249; (41,102) ,0.00088249;
(42,102) ,0.00088249; (44,102) ,0.00088249; (46,102) ,0.00088249; (48,102) ,0.00088249; (49,102)
,0.00088249; (50,102) ,0.00088249; (51,102) ,0.00088249; (52,102) ,0.00088249; (54,102) ,0.00088249;
(57,102) ,0.00088249; (63,102) ,0.00088249; (64,102) ,0.00088249; (67,102) ,0.00088249; (69,102)
,0.00088249; (70,102) ,0.00088249; (73,102) ,0.00088249; (75,102) ,0.00088249; (78,102) ,0.00088249;
(79,102) ,0.00088249; (84,102) ,0.00088249; (85,102) ,0.00088249; (86,102) ,0.00088249; (87,102)
,0.00088249; (97,102) ,0.00088249; (107,102) ,0.00088249; (108,102) ,0.00088249; (112,102)
,0.00088249; (114,102) ,0.00088249; (115,102) ,0.00088249; (119,102) ,0.00088249; (122,102)
,0.00088249; (132,102) ,0.00088249; (133,102) ,0.00088249; (135,102) ,0.00088249; (136,102)
,0.00088249; (138,102) ,0.00088249; (142,102) ,0.00088249; (143,102) ,0.00088249; (2,103) ,3.81e-08;
(3,103) ,3.81e-08; (4,103) ,3.81e-08; (5,103) ,3.81e-08; (6,103) ,3.81e-08; (7,103) ,3.81e-08;
(8,103) ,3.81e-08; (9,103) ,3.81e-08; (10,103) ,3.81e-08; (11,103) ,3.81e-08; (12,103) ,3.81e-08;
(13,103) ,3.81e-08; (14,103) ,3.81e-08; (15,103) ,3.81e-08; (16,103) ,3.81e-08; (19,103) ,3.81e-08;
(20,103) ,3.81e-08; (21,103) ,3.81e-08; (22,103) ,3.81e-08; (23,103) ,3.81e-08; (24,103) ,3.81e-08;
(26,103) ,3.81e-08; (27,103) ,3.81e-08; (28,103) ,3.81e-08; (29,103) ,3.81e-08; (30,103) ,3.81e-08;
(31,103) ,3.81e-08; (32,103) ,3.81e-08; (33,103) ,3.81e-08; (34,103) ,3.81e-08; (35,103) ,3.81e-08;
(36,103) ,3.81e-08; (37,103) ,3.81e-08; (38,103) ,3.81e-08; (39,103) ,3.81e-08; (41,103) ,3.81e-08;
(42,103) ,3.81e-08; (44,103) ,3.81e-08; (46,103) ,3.81e-08; (48,103) ,3.81e-08; (49,103) ,3.81e-08;
(50,103) ,3.81e-08; (51,103) ,3.81e-08; (52,103) ,3.81e-08; (54,103) ,3.81e-08; (57,103) ,3.81e-08;
(63,103) ,3.81e-08; (64,103) ,3.81e-08; (67,103) ,3.81e-08; (69,103) ,3.81e-08; (70,103) ,3.81e-08;
(73,103) ,3.81e-08; (75,103) ,3.81e-08; (78,103) ,3.81e-08; (79,103) ,3.81e-08; (84,103) ,3.81e-08;
(85,103) ,3.81e-08; (86,103) ,3.81e-08; (87,103) ,3.81e-08; (97,103) ,3.81e-08; (107,103) ,3.81e-08;
(108,103) ,3.81e-08; (112,103) ,3.81e-08; (114,103) ,3.81e-08; (115,103) ,3.81e-08; (119,103)
,3.81e-08; (122,103) ,3.81e-08; (132,103) ,3.81e-08; (133,103) ,3.81e-08; (135,103) ,3.81e-08;
(136,103) ,3.81e-08; (138,103) ,3.81e-08; (142,103) ,3.81e-08; (143,103) ,3.81e-08; (2,105)
,0.00401379; (3,105) ,0.00401379; (4,105) ,0.00401379; (5,105) ,0.00401379; (13,105) ,0.00401379;
(28,105) ,0.00401379; (2,106) ,0.00305724; (3,106) ,0.00305724; (4,106) ,0.00305724; (5,106)
,0.00305724; (6,106) ,0.00305724; (8,106) ,0.00305724; (10,106) ,0.00305724; (13,106) ,0.00305724;
(14,106) ,0.00305724; (19,106) ,0.00305724; (22,106) ,0.00305724; (24,106) ,0.00305724; (28,106)
,0.00305724; (30,106) ,0.00305724; (33,106) ,0.00305724; (35,106) ,0.00305724;

(36,106) ,0.00305724;
(37,106) ,0.00305724; (38,106) ,0.00305724; (39,106) ,0.00305724; (49,106) ,0.00305724; (52,106)
,0.00305724; (64,106) ,0.00305724; (69,106) ,0.00305724; (86,106) ,0.00305724; (119,106) ,0.00305724;
(132,106) ,0.00305724; (138,106) ,0.00305724; (142,106) ,0.00305724; (143,106) ,0.00305724; (2,108)
,0.0049509; (3,108) ,0.0049509; (4,108) ,0.0049509; (5,108) ,0.0049509; (7,108) ,0.0049509; (10,108)
,0.0049509; (11,108) ,0.0049509; (13,108) ,0.0049509; (28,108) ,0.0049509; (33,108) ,0.0049509;
(36,108) ,0.0049509; (37,108) ,0.0049509; (38,108) ,0.0049509; (69,108) ,0.0049509; (86,108)
,0.0049509; (138,108) ,0.0049509; (143,108) ,0.0049509; (2,109) ,0.0018609; (3,109) ,0.0018609;
(4,109) ,0.0018609; (5,109) ,0.0018609; (6,109) ,0.0018609; (7,109) ,0.0018609; (9,109) ,0.0018609;
(10,109) ,0.0018609; (11,109) ,0.0018609; (12,109) ,0.0018609; (13,109) ,0.0018609; (14,109)
,0.0018609; (15,109) ,0.0018609; (16,109) ,0.0018609; (19,109) ,0.0018609; (21,109) ,0.0018609;
(22,109) ,0.0018609; (23,109) ,0.0018609; (24,109) ,0.0018609; (26,109) ,0.0018609; (28,109)
,0.0018609; (29,109) ,0.0018609; (30,109) ,0.0018609; (31,109) ,0.0018609; (32,109) ,0.0018609;
(33,109) ,0.0018609; (35,109) ,0.0018609; (36,109) ,0.0018609; (37,109) ,0.0018609; (38,109)
,0.0018609; (39,109) ,0.0018609; (41,109) ,0.0018609; (42,109) ,0.0018609; (46,109) ,0.0018609;
(49,109) ,0.0018609; (50,109) ,0.0018609; (52,109) ,0.0018609; (54,109) ,0.0018609; (57,109)
,0.0018609; (64,109) ,0.0018609; (67,109) ,0.0018609; (69,109) ,0.0018609; (70,109) ,0.0018609;
(75,109) ,0.0018609; (86,109) ,0.0018609; (97,109) ,0.0018609; (107,109) ,0.0018609; (108,109)
,0.0018609; (114,109) ,0.0018609; (115,109) ,0.0018609; (119,109) ,0.0018609; (133,109) ,0.0018609;
(138,109) ,0.0018609; (142,109) ,0.0018609; (143,109) ,0.0018609; (2,111) ,0.00419724; (3,111)
,0.00419724; (4,111) ,0.00419724; (5,111) ,0.00419724; (6,111) ,0.00419724; (7,111) ,0.00419724;
(14,111) ,0.00419724; (22,111) ,0.00419724; (24,111) ,0.00419724; (28,111) ,0.00419724; (33,111)
,0.00419724; (36,111) ,0.00419724; (38,111) ,0.00419724; (39,111) ,0.00419724; (49,111) ,0.00419724;
(64,111) ,0.00419724; (86,111) ,0.00419724; (119,111) ,0.00419724; (132,111) ,0.00419724; (138,111)
,0.00419724; (2,112) ,0.00122254; (3,112) ,0.00122254; (4,112) ,0.00122254; (5,112) ,0.00122254;
(6,112) ,0.00122254; (7,112) ,0.00122254; (8,112) ,0.00122254; (9,112) ,0.00122254; (10,112)
,0.00122254; (12,112) ,0.00122254; (13,112) ,0.00122254; (14,112) ,0.00122254; (15,112) ,0.00122254;
(16,112) ,0.00122254; (20,112) ,0.00122254; (21,112) ,0.00122254; (22,112) ,0.00122254; (24,112)
,0.00122254; (26,112) ,0.00122254; (27,112) ,0.00122254; (28,112) ,0.00122254; (29,112) ,0.00122254;
(30,112) ,0.00122254; (32,112) ,0.00122254; (33,112) ,0.00122254; (34,112) ,0.00122254;

(35,112)
,0.00122254; (36,112) ,0.00122254; (37,112) ,0.00122254; (38,112) ,0.00122254; (39,112) ,0.00122254;
(42,112) ,0.00122254; (44,112) ,0.00122254; (46,112) ,0.00122254; (48,112) ,0.00122254; (49,112)
,0.00122254; (50,112) ,0.00122254; (51,112) ,0.00122254; (52,112) ,0.00122254; (54,112) ,0.00122254;
(63,112) ,0.00122254; (64,112) ,0.00122254; (67,112) ,0.00122254; (69,112) ,0.00122254; (70,112)
8
(63,112) ,0.00122254; (64,112) ,0.00122254; (67,112) ,0.00122254; (69,112) ,0.00122254; (70,112)
,0.00122254; (73,112) ,0.00122254; (75,112) ,0.00122254; (78,112) ,0.00122254; (79,112) ,0.00122254;
(84,112) ,0.00122254; (85,112) ,0.00122254; (86,112) ,0.00122254; (87,112) ,0.00122254; (107,112)
,0.00122254; (108,112) ,0.00122254; (112,112) ,0.00122254; (114,112) ,0.00122254; (119,112)
,0.00122254; (122,112) ,0.00122254; (132,112) ,0.00122254; (135,112) ,0.00122254; (136,112)
,0.00122254; (138,112) ,0.00122254; (142,112) ,0.00122254; (143,112) ,0.00122254; (2,114)
,0.00430556; (3,114) ,0.00430556; (4,114) ,0.00430556; (5,114) ,0.00430556; (6,114) ,0.00430556;
(36,114) ,0.00430556; (138,114) ,0.00430556; (2,115) ,0.0028616; (3,115) ,0.0028616; (4,115)
,0.0028616; (5,115) ,0.0028616; (6,115) ,0.0028616; (7,115) ,0.0028616; (11,115) ,0.0028616; (12,115)
,0.0028616; (13,115) ,0.0028616; (14,115) ,0.0028616; (15,115) ,0.0028616; (21,115) ,0.0028616;
(24,115) ,0.0028616; (26,115) ,0.0028616; (28,115) ,0.0028616; (29,115) ,0.0028616; (32,115)
,0.0028616; (33,115) ,0.0028616; (35,115) ,0.0028616; (36,115) ,0.0028616; (37,115) ,0.0028616;
(38,115) ,0.0028616; (42,115) ,0.0028616; (46,115) ,0.0028616; (49,115) ,0.0028616; (50,115)
,0.0028616; (52,115) ,0.0028616; (54,115) ,0.0028616; (69,115) ,0.0028616; (70,115) ,0.0028616;
(84,115) ,0.0028616; (86,115) ,0.0028616; (119,115) ,0.0028616; (138,115) ,0.0028616; (142,115)
,0.0028616; (143,115) ,0.0028616; (2,136) ,3e+08; (3,136) ,3e+08; (4,136) ,3e+08; (5,136) ,3e+08;
(6,136) ,3e+08; (7,136) ,3e+08; (10,136) ,3e+08; (11,136) ,3e+08; (13,136) ,3e+08; (14,136) ,3e+08;
(22,136) ,3e+08; (24,136) ,3e+08; (28,136) ,3e+08; (33,136) ,3e+08; (36,136) ,3e+08; (37,136) ,3e+08;
(38,136) ,3e+08; (39,136) ,3e+08; (49,136) ,3e+08; (64,136) ,3e+08; (69,136) ,3e+08; (86,136) ,3e+08;
(119,136) ,3e+08; (132,136) ,3e+08; (138,136) ,3e+08; (143,136) ,3e+08; (2,137) ,9.84e+14; (3,137)
,9.84e+14; (4,137) ,9.84e+14; (5,137) ,9.84e+14; (6,137) ,9.84e+14; (7,137) ,9.84e+14; (10,137)
,9.84e+14; (11,137) ,9.84e+14; (13,137) ,9.84e+14; (14,137) ,9.84e+14; (22,137) ,9.84e+14; (24,137)
,9.84e+14; (28,137) ,9.84e+14; (33,137) ,9.84e+14; (36,137) ,9.84e+14; (37,137) ,9.84e+14;

(38,137)
,9.84e+14; (39,137) ,9.84e+14; (49,137) ,9.84e+14; (64,137) ,9.84e+14; (69,137) ,9.84e+14; (86,137)
,9.84e+14; (119,137) ,9.84e+14; (132,137) ,9.84e+14; (138,137) ,9.84e+14; (143,137) ,9.84e+14;
(2,138) ,3.05e+08; (3,138) ,3.05e+08; (4,138) ,3.05e+08; (5,138) ,3.05e+08; (6,138) ,3.05e+08;
(7,138) ,3.05e+08; (8,138) ,3.05e+08; (9,138) ,3.05e+08; (10,138) ,3.05e+08; (11,138) ,3.05e+08;
(12,138) ,3.05e+08; (13,138) ,3.05e+08; (14,138) ,3.05e+08; (15,138) ,3.05e+08; (16,138) ,3.05e+08;
(19,138) ,3.05e+08; (20,138) ,3.05e+08; (21,138) ,3.05e+08; (22,138) ,3.05e+08; (23,138) ,3.05e+08;
(24,138) ,3.05e+08; (26,138) ,3.05e+08; (27,138) ,3.05e+08; (28,138) ,3.05e+08; (29,138) ,3.05e+08;
(30,138) ,3.05e+08; (31,138) ,3.05e+08; (32,138) ,3.05e+08; (33,138) ,3.05e+08; (34,138) ,3.05e+08;
(35,138) ,3.05e+08; (36,138) ,3.05e+08; (37,138) ,3.05e+08; (38,138) ,3.05e+08; (39,138) ,3.05e+08;
(41,138) ,3.05e+08; (42,138) ,3.05e+08; (44,138) ,3.05e+08; (46,138) ,3.05e+08; (48,138) ,3.05e+08;
(49,138) ,3.05e+08; (50,138) ,3.05e+08; (51,138) ,3.05e+08; (52,138) ,3.05e+08; (54,138) ,3.05e+08;
(57,138) ,3.05e+08; (63,138) ,3.05e+08; (64,138) ,3.05e+08; (67,138) ,3.05e+08; (69,138) ,3.05e+08;
(70,138) ,3.05e+08; (73,138) ,3.05e+08; (75,138) ,3.05e+08; (78,138) ,3.05e+08; (79,138) ,3.05e+08;
(84,138) ,3.05e+08; (85,138) ,3.05e+08; (86,138) ,3.05e+08; (87,138) ,3.05e+08; (97,138) ,3.05e+08;
(107,138) ,3.05e+08; (108,138) ,3.05e+08; (112,138) ,3.05e+08; (114,138) ,3.05e+08; (115,138)
,3.05e+08; (119,138) ,3.05e+08; (122,138) ,3.05e+08; (132,138) ,3.05e+08; (133,138) ,3.05e+08;
(135,138) ,3.05e+08; (136,138) ,3.05e+08; (138,138) ,3.05e+08; (142,138) ,3.05e+08; (143,138)
,3.05e+08; (2,139) ,5.8e+14; (3,139) ,5.8e+14; (4,139) ,5.8e+14; (5,139) ,5.8e+14; (6,139) ,5.8e+14;
(7,139) ,5.8e+14; (8,139) ,5.8e+14; (9,139) ,5.8e+14; (10,139) ,5.8e+14; (11,139) ,5.8e+14; (12,139)
,5.8e+14; (13,139) ,5.8e+14; (14,139) ,5.8e+14; (15,139) ,5.8e+14; (16,139) ,5.8e+14; (19,139)
,5.8e+14; (20,139) ,5.8e+14; (21,139) ,5.8e+14; (22,139) ,5.8e+14; (23,139) ,5.8e+14; (24,139)
,5.8e+14; (26,139) ,5.8e+14; (27,139) ,5.8e+14; (28,139) ,5.8e+14; (29,139) ,5.8e+14; (30,139)
,5.8e+14; (31,139) ,5.8e+14; (32,139) ,5.8e+14; (33,139) ,5.8e+14; (34,139) ,5.8e+14; (35,139)
,5.8e+14; (36,139) ,5.8e+14; (37,139) ,5.8e+14; (38,139) ,5.8e+14; (39,139) ,5.8e+14; (41,139)
,5.8e+14; (42,139) ,5.8e+14; (44,139) ,5.8e+14; (46,139) ,5.8e+14; (48,139) ,5.8e+14; (49,139)
,5.8e+14; (50,139) ,5.8e+14; (51,139) ,5.8e+14; (52,139) ,5.8e+14; (54,139) ,5.8e+14; (57,139)
,5.8e+14; (63,139) ,5.8e+14; (64,139) ,5.8e+14; (67,139) ,5.8e+14; (69,139) ,5.8e+14; (70,139)
,5.8e+14; (73,139) ,5.8e+14; (75,139) ,5.8e+14; (78,139) ,5.8e+14; (79,139) ,5.8e+14; (84,139)
,5.8e+14; (85,139) ,5.8e+14; (86,139) ,5.8e+14; (87,139) ,5.8e+14; (97,139) ,5.8e+14; (107,139)
,5.8e+14; (108,139) ,5.8e+14; (112,139) ,5.8e+14; (114,139) ,5.8e+14; (115,139) ,5.8e+14;

(119,139)
,5.8e+14; (122,139) ,5.8e+14; (132,139) ,5.8e+14; (133,139) ,5.8e+14; (135,139) ,5.8e+14; (136,139)
,5.8e+14; (138,139) ,5.8e+14; (142,139) ,5.8e+14; (143,139) ,5.8e+14; (2,141) ,3.02e+08; (3,141)
,3.02e+08; (4,141) ,3.02e+08; (5,141) ,3.02e+08; (13,141) ,3.02e+08; (28,141) ,3.02e+08; (2,142)
,2.62e+08; (3,142) ,2.62e+08; (4,142) ,2.62e+08; (5,142) ,2.62e+08; (6,142) ,2.62e+08; (8,142)
,2.62e+08; (10,142) ,2.62e+08; (13,142) ,2.62e+08; (14,142) ,2.62e+08; (19,142) ,2.62e+08; (22,142)
,2.62e+08; (24,142) ,2.62e+08; (28,142) ,2.62e+08; (30,142) ,2.62e+08; (33,142) ,2.62e+08; (35,142)
,2.62e+08; (36,142) ,2.62e+08; (37,142) ,2.62e+08; (38,142) ,2.62e+08; (39,142) ,2.62e+08; (49,142)
,2.62e+08; (52,142) ,2.62e+08; (64,142) ,2.62e+08; (69,142) ,2.62e+08; (86,142) ,2.62e+08; (119,142)
,2.62e+08; (132,142) ,2.62e+08; (138,142) ,2.62e+08; (142,142) ,2.62e+08; (143,142) ,2.62e+08;
(2,144) ,2.61e+08; (3,144) ,2.61e+08; (4,144) ,2.61e+08; (5,144) ,2.61e+08; (7,144) ,2.61e+08;
(10,144) ,2.61e+08; (11,144) ,2.61e+08; (13,144) ,2.61e+08; (28,144) ,2.61e+08; (33,144) ,2.61e+08;
(36,144) ,2.61e+08; (37,144) ,2.61e+08; (38,144) ,2.61e+08; (69,144) ,2.61e+08; (86,144) ,2.61e+08;
(138,144) ,2.61e+08; (143,144) ,2.61e+08; (2,145) ,2.52e+08; (3,145) ,2.52e+08; (4,145) ,2.52e+08;
(5,145) ,2.52e+08; (6,145) ,2.52e+08; (7,145) ,2.52e+08; (9,145) ,2.52e+08; (10,145) ,2.52e+08;
(11,145) ,2.52e+08; (12,145) ,2.52e+08; (13,145) ,2.52e+08; (14,145) ,2.52e+08; (15,145) ,2.52e+08;

(11,145) ,2.52e+08; (12,145) ,2.52e+08; (13,145) ,2.52e+08; (14,145) ,2.52e+08; (15,145) ,2.52e+08;
(16,145) ,2.52e+08; (19,145) ,2.52e+08; (21,145) ,2.52e+08; (22,145) ,2.52e+08; (23,145) ,2.52e+08;
(24,145) ,2.52e+08; (26,145) ,2.52e+08; (28,145) ,2.52e+08; (29,145) ,2.52e+08; (30,145) ,2.52e+08;
(31,145) ,2.52e+08; (32,145) ,2.52e+08; (33,145) ,2.52e+08; (35,145) ,2.52e+08; (36,145) ,2.52e+08;
(37,145) ,2.52e+08; (38,145) ,2.52e+08; (39,145) ,2.52e+08; (41,145) ,2.52e+08; (42,145) ,2.52e+08;
(46,145) ,2.52e+08; (49,145) ,2.52e+08; (50,145) ,2.52e+08; (52,145) ,2.52e+08; (54,145) ,2.52e+08;
(57,145) ,2.52e+08; (64,145) ,2.52e+08; (67,145) ,2.52e+08; (69,145) ,2.52e+08; (70,145) ,2.52e+08;
(75,145) ,2.52e+08; (86,145) ,2.52e+08; (97,145) ,2.52e+08; (107,145) ,2.52e+08; (108,145) ,2.52e+08;
(114,145) ,2.52e+08; (115,145) ,2.52e+08; (119,145) ,2.52e+08; (133,145) ,2.52e+08; (138,145)
,2.52e+08; (142,145) ,2.52e+08; (143,145) ,2.52e+08; (2,147) ,2.56e+08; (3,147) ,2.56e+08;

(4,147)
,2.56e+08; (5,147) ,2.56e+08; (6,147) ,2.56e+08; (7,147) ,2.56e+08; (14,147) ,2.56e+08; (22,147)
,2.56e+08; (24,147) ,2.56e+08; (28,147) ,2.56e+08; (33,147) ,2.56e+08; (36,147) ,2.56e+08;
(38,147)
,2.56e+08; (39,147) ,2.56e+08; (49,147) ,2.56e+08; (64,147) ,2.56e+08; (86,147) ,2.56e+08;
(119,147)
,2.56e+08; (132,147) ,2.56e+08; (138,147) ,2.56e+08; (2,148) ,2.58e+08; (3,148) ,2.58e+08;
(4,148)
,2.58e+08; (5,148) ,2.58e+08; (6,148) ,2.58e+08; (7,148) ,2.58e+08; (8,148) ,2.58e+08; (9,148)
,2.58e+08; (10,148) ,2.58e+08; (12,148) ,2.58e+08; (13,148) ,2.58e+08; (14,148) ,2.58e+08;
(15,148)
,2.58e+08; (16,148) ,2.58e+08; (20,148) ,2.58e+08; (21,148) ,2.58e+08; (22,148) ,2.58e+08;
(24,148)
,2.58e+08; (26,148) ,2.58e+08; (27,148) ,2.58e+08; (28,148) ,2.58e+08; (29,148) ,2.58e+08;
(30,148)
,2.58e+08; (32,148) ,2.58e+08; (33,148) ,2.58e+08; (34,148) ,2.58e+08; (35,148) ,2.58e+08;
(36,148)
,2.58e+08; (37,148) ,2.58e+08; (38,148) ,2.58e+08; (39,148) ,2.58e+08; (42,148) ,2.58e+08;
(44,148)
,2.58e+08; (46,148) ,2.58e+08; (48,148) ,2.58e+08; (49,148) ,2.58e+08; (50,148) ,2.58e+08;
(51,148)
,2.58e+08; (52,148) ,2.58e+08; (54,148) ,2.58e+08; (63,148) ,2.58e+08; (64,148) ,2.58e+08;
(67,148)
,2.58e+08; (69,148) ,2.58e+08; (70,148) ,2.58e+08; (73,148) ,2.58e+08; (75,148) ,2.58e+08;
(78,148)
,2.58e+08; (79,148) ,2.58e+08; (84,148) ,2.58e+08; (85,148) ,2.58e+08; (86,148) ,2.58e+08;
(87,148)
,2.58e+08; (107,148) ,2.58e+08; (108,148) ,2.58e+08; (112,148) ,2.58e+08; (114,148) ,2.58e+08;
(119,148) ,2.58e+08; (122,148) ,2.58e+08; (132,148) ,2.58e+08; (135,148) ,2.58e+08; (136,148)
,2.58e+08; (138,148) ,2.58e+08; (142,148) ,2.58e+08; (143,148) ,2.58e+08; (2,150) ,2.83e+08;
(3,150)
,2.83e+08; (4,150) ,2.83e+08; (5,150) ,2.83e+08; (6,150) ,2.83e+08; (36,150) ,2.83e+08; (138,150)
,2.83e+08; (2,151) ,2.48e+08; (3,151) ,2.48e+08; (4,151) ,2.48e+08; (5,151) ,2.48e+08; (6,151)
,2.48e+08; (7,151) ,2.48e+08; (11,151) ,2.48e+08; (12,151) ,2.48e+08; (13,151) ,2.48e+08;
(14,151)
,2.48e+08; (15,151) ,2.48e+08; (21,151) ,2.48e+08; (24,151) ,2.48e+08; (26,151) ,2.48e+08;
(28,151)
,2.48e+08; (29,151) ,2.48e+08; (32,151) ,2.48e+08; (33,151) ,2.48e+08; (35,151) ,2.48e+08;
(36,151)
,2.48e+08; (37,151) ,2.48e+08; (38,151) ,2.48e+08; (42,151) ,2.48e+08; (46,151) ,2.48e+08;
(49,151)
,2.48e+08; (50,151) ,2.48e+08; (52,151) ,2.48e+08; (54,151) ,2.48e+08; (69,151) ,2.48e+08;
(70,151)
,2.48e+08; (84,151) ,2.48e+08; (86,151) ,2.48e+08; (119,151) ,2.48e+08; (138,151) ,2.48e+08;

(142,151) ,2.48e+08; (143,151) ,2.48e+08; (2,154) ,1361; (3,154) ,1361; (4,154) ,1361; (5,154) ,1361;
(6,154) ,1361; (7,154) ,1361; (10,154) ,1361; (11,154) ,1361; (13,154) ,1361; (14,154) ,1361; (22,154) ,1361; (24,154) ,1361; (28,154) ,1361; (33,154) ,1361; (36,154) ,1361; (37,154) ,1361; (38,154) ,1361; (39,154) ,1361; (49,154) ,1361; (64,154) ,1361; (69,154) ,1361; (86,154) ,1361; (119,154) ,1361; (132,154) ,1361; (138,154) ,1361; (143,154) ,1361; (2,155) ,52691.6; (3,155) ,52691.6; (4,155) ,52691.6; (5,155) ,52691.6; (6,155) ,52691.6; (7,155) ,52691.6; (10,155) ,52691.6;
(11,155) ,52691.6; (13,155) ,52691.6; (14,155) ,52691.6; (22,155) ,52691.6; (24,155) ,52691.6; (28,155) ,52691.6; (33,155) ,52691.6; (36,155) ,52691.6; (37,155) ,52691.6; (38,155) ,52691.6; (39,155) ,52691.6; (49,155) ,52691.6; (64,155) ,52691.6; (69,155) ,52691.6; (86,155) ,52691.6; (119,155) ,52691.6; (132,155) ,52691.6; (138,155) ,52691.6; (143,155) ,52691.6; (2,156) ,1416.05;
(3,156) ,1416.05; (4,156) ,1416.05; (5,156) ,1416.05; (6,156) ,1416.05; (7,156) ,1416.05; (8,156) ,1416.05; (9,156) ,1416.05; (10,156) ,1416.05; (11,156) ,1416.05; (12,156) ,1416.05; (13,156) ,1416.05; (14,156) ,1416.05; (15,156) ,1416.05; (16,156) ,1416.05; (19,156) ,1416.05; (20,156) ,1416.05; (21,156) ,1416.05; (22,156) ,1416.05; (23,156) ,1416.05; (24,156) ,1416.05; (26,156) ,1416.05; (27,156) ,1416.05; (28,156) ,1416.05; (29,156) ,1416.05; (30,156) ,1416.05; (31,156) ,1416.05; (32,156) ,1416.05; (33,156) ,1416.05; (34,156) ,1416.05; (35,156) ,1416.05; (36,156) ,1416.05; (37,156) ,1416.05; (38,156) ,1416.05; (39,156) ,1416.05; (41,156) ,1416.05; (42,156) ,1416.05; (44,156) ,1416.05; (46,156) ,1416.05; (48,156) ,1416.05; (49,156) ,1416.05; (50,156) ,1416.05; (51,156) ,1416.05; (52,156) ,1416.05; (54,156) ,1416.05; (57,156) ,1416.05; (63,156) ,1416.05; (64,156) ,1416.05; (67,156) ,1416.05; (69,156) ,1416.05; (70,156) ,1416.05; (73,156) ,1416.05; (75,156) ,1416.05; (78,156) ,1416.05; (79,156) ,1416.05; (84,156) ,1416.05; (85,156) ,1416.05; (86,156) ,1416.05; (87,156) ,1416.05; (97,156) ,1416.05; (107,156) ,1416.05; (108,156) ,1416.05; (112,156) ,1416.05; (114,156) ,1416.05; (115,156) ,1416.05; (119,156) ,1416.05; (122,156)
,1416.05; (132,156) ,1416.05; (133,156) ,1416.05; (135,156) ,1416.05; (136,156) ,1416.05; (138,156)
,1416.05; (142,156) ,1416.05; (143,156) ,1416.05; (2,157) ,31502.8; (3,157) ,31502.8; (4,157) ,31502.8; (5,157) ,31502.8; (6,157) ,31502.8; (7,157) ,31502.8; (8,157) ,31502.8; (9,157) ,31502.8; (10,157) ,31502.8; (11,157) ,31502.8; (12,157) ,31502.8; (13,157) ,31502.8; (14,157) ,31502.8; (15,157) ,31502.8; (16,157) ,31502.8; (19,157) ,31502.8; (20,157) ,31502.8; (21,157) ,31502.8; (22,157) ,31502.8; (23,157) ,31502.8; (24,157) ,31502.8; (26,157) ,31502.8; (27,157) ,31502.8; (28,157) ,31502.8; (29,157) ,31502.8; (30,157) ,31502.8; (31,157) ,31502.8; (32,157) ,31502.8; (33,157) ,31502.8; (34,157) ,31502.8; (35,157) ,31502.8; (36,157) ,31502.8; (37,157) ,31502.8; (38,157) ,31502.8; (39,157) ,31502.8; (41,157) ,31502.8; (42,157) ,31502.8; (44,157) ,31502.8; (46,157) ,31502.8; (48,157) ,31502.8; (49,157) ,31502.8; (50,157) ,31502.8; (51,157) ,31502.8;

10

(46,157) ,31502.8; (48,157) ,31502.8; (49,157) ,31502.8; (50,157) ,31502.8; (51,157) ,31502.8;
(52,157) ,31502.8; (54,157) ,31502.8; (57,157) ,31502.8; (63,157) ,31502.8; (64,157) ,31502.8; (67,157) ,31502.8; (69,157) ,31502.8; (70,157) ,31502.8; (73,157) ,31502.8; (75,157) ,31502.8; (78,157) ,31502.8; (79,157) ,31502.8; (84,157) ,31502.8; (85,157) ,31502.8; (86,157) ,31502.8; (87,157) ,31502.8; (97,157) ,31502.8; (107,157) ,31502.8; (108,157) ,31502.8;

(112,157) ,31502.8;
(114,157) ,31502.8; (115,157) ,31502.8; (119,157) ,31502.8; (122,157) ,31502.8; (132,157) ,31502.8;
(133,157) ,31502.8; (135,157) ,31502.8; (136,157) ,31502.8; (138,157) ,31502.8; (142,157) ,31502.8;
(143,157) ,31502.8; (2,159) ,1186.8; (3,159) ,1186.8; (4,159) ,1186.8; (,1186.8; (28,159) ,1186.8; (2,160) ,1455.35; (3,160) ,1455.35; (4,160) ,1455.35; (5,160) ,1455.35;
(6,160) ,1455.35; (8,160) ,1455.35; (10,160) ,1455.35; (13,160) ,1455.35; (14,160) ,1455.35; (19,160)
,1455.35; (22,160) ,1455.35; (24,160) ,1455.35; (28,160) ,1455.35; (30,160) ,1455.35; (33,160) ,1455.35; (35,160) ,1455.35; (36,160) ,1455.35; (37,160) ,1455.35; (38,160) ,1455.35; (39,160) ,1455.35; (49,160) ,1455.35; (52,160) ,1455.35; (64,160) ,1455.35; (69,160) ,1455.35; (86,160) ,1455.35; (119,160) ,1455.35; (132,160) ,1455.35; (138,160) ,1455.35; (142,160) ,1455.35; (143,160)
,1455.35; (2,162) ,1399.9; (3,162) ,1399.9; (4,162) ,1399.9; (5,162) ,1399.9; (7,162) ,1399.9; (10,162) ,1399.9; (11,162) ,1399.9; (13,162) ,1399.9; (28,162) ,1399.9; (33,162) ,1399.9; (36,162) ,1399.9; (37,162) ,1399.9; (38,162) ,1399.9; (69,162) ,1399.9; (86,162) ,1399.9; (138,162) ,1399.9; (143,162) ,1399.9; (2,163) ,1614.78; (3,163) ,1614.78; (4,163) ,1614.78; (5,163) ,1614.78; (6,163) ,1614.78; (7,163) ,1614.78; (9,163) ,1614.78; (10,163) ,1614.78; (11,163) ,1614.78; (12,163) ,1614.78; (13,163) ,1614.78; (14,163) ,1614.78; (15,163) ,1614.78; (16,163) ,1614.78; (19,163) ,1614.78; (21,163) ,1614.78; (22,163) ,1614.78; (23,163) ,1614.78; (24,163) ,1614.78; (26,163) ,1614.78; (28,163) ,1614.78; (29,163) ,1614.78; (30,163) ,1614.78; (31,163) ,1614.78; (32,163) ,1614.78; (33,163) ,1614.78; (35,163) ,1614.78; (36,163) ,1614.78; (37,163) ,1614.78; (38,163) ,1614.78; (39,163) ,1614.78; (41,163) ,1614.78; (42,163) ,1614.78; (46,163) ,1614.78; (49,163) ,1614.78; (50,163) ,1614.78; (52,163) ,1614.78; (54,163) ,1614.78; (57,163) ,1614.78; (64,163) ,1614.78; (67,163) ,1614.78; (69,163) ,1614.78; (70,163) ,1614.78; (75,163) ,1614.78; (86,163) ,1614.78; (97,163) ,1614.78; (107,163) ,1614.78; (108,163) ,1614.78; (114,163) ,1614.78; (115,163)
,1614.78; (119,163) ,1614.78; (133,163) ,1614.78; (138,163) ,1614.78; (142,163) ,1614.78; (143,163)
,1614.78; (2,165) ,1438.7; (3,165) ,1438.7; (4,165) ,1438.7; (5,165) ,1438.7; (6,165) ,1438.7; (7,165) ,1438.7; (14,165) ,1438.7; (22,165) ,1438.7; (24,165) ,1438.7; (28,165) ,1438.7; (33,165) ,1438.7; (36,165) ,1438.7; (38,165) ,1438.7; (39,165) ,1438.7; (49,165) ,1438.7; (64,165) ,1438.7; (86,165) ,1438.7; (119,165) ,1438.7; (132,165) ,1438.7; (138,165) ,1438.7; (2,166) ,1455; (3,166) ,1455; (4,166) ,1455; (5,166) ,1455; (6,166) ,1455; (7,166) ,1455; (8,166) ,1455; (9,166) ,1455; (10,166) ,1455; (12,166) ,1455; (13,166) ,1455; (14,166) ,1455; (15,166) ,1455; (16,166) ,1455; (20,166) ,1455; (21,166) ,1455; (22,166) ,1455; (24,166) ,1455; (26,166) ,1455; (27,166) ,1455; (28,166) ,1455; (29,166) ,1455; (30,166) ,1455; (32,166) ,1455; (33,166) ,1455; (34,166) ,1455; (35,166) ,1455; (36,166) ,1455; (37,166) ,1455; (38,166) ,1455; (39,166) ,1455; (42,166) ,1455; (44,166) ,1455; (46,166) ,1455; (48,166) ,1455; (49,166) ,1455; (50,166) ,1455; (51,166) ,1455; (52,166) ,1455; (54,166) ,1455; (63,166) ,1455; (64,166) ,1455; (67,166) ,1455; (69,166) ,1455; (70,166) ,1455; (73,166) ,1455; (75,166) ,1455; (78,166) ,1455; (79,166) ,1455; (84,166) ,1455; (85,166) ,1455; (86,166) ,1455; (87,166) ,1455; (107,166) ,1455; (108,166) ,1455; (112,166) ,1455;

(114,166) ,1455; (119,166) ,1455; (122,166) ,1455; (132,166) ,1455; (135,166) ,1455;
(136,166) ,1455;
(138,166) ,1455; (142,166) ,1455; (143,166) ,1455; (2,168) ,1278.34; (3,168) ,1278.34; (4,168)
,1278.34; (5,168) ,1278.34; (6,168) ,1278.34; (36,168) ,1278.34; (138,168) ,1278.34; (2,169)
,1385.47; (3,169) ,1385.47; (4,169) ,1385.47; (5,169) ,1385.47; (6,169) ,1385.47; (7,169) ,1385.47;
(11,169) ,1385.47; (12,169) ,1385.47; (13,169) ,1385.47; (14,169) ,1385.47; (15,169) ,1385.47;
(21,169) ,1385.47; (24,169) ,1385.47; (26,169) ,1385.47; (28,169) ,1385.47; (29,169) ,1385.47;
(32,169) ,1385.47; (33,169) ,1385.47; (35,169) ,1385.47; (36,169) ,1385.47; (37,169) ,1385.47;
(38,169) ,1385.47; (42,169) ,1385.47; (46,169) ,1385.47; (49,169) ,1385.47; (50,169) ,1385.47;
(52,169) ,1385.47; (54,169) ,1385.47; (69,169) ,1385.47; (70,169) ,1385.47; (84,169) ,1385.47;
(86,169) ,1385.47; (119,169) ,1385.47; (138,169) ,1385.47; (142,169) ,1385.47;
(143,169) ,1385.47;
(2,172) ,0.0374128; (3,172) ,0.0374128; (4,172) ,0.0374128; (5,172) ,0.0374128;
(6,172) ,0.0374128;
(7,172) ,0.0374128; (10,172) ,0.0374128; (11,172) ,0.0374128; (13,172) ,0.0374128; (14,172)
,0.0374128; (22,172) ,0.0374128; (24,172) ,0.0374128; (28,172) ,0.0374128; (33,172) ,0.0374128;
(36,172) ,0.0374128; (37,172) ,0.0374128; (38,172) ,0.0374128; (39,172) ,0.0374128; (49,172)
,0.0374128; (64,172) ,0.0374128; (69,172) ,0.0374128; (86,172) ,0.0374128;
(119,172) ,0.0374128;
(132,172) ,0.0374128; (138,172) ,0.0374128; (143,172) ,0.0374128; (2,173) ,6.04e-05; (3,173)
,6.04e-05; (4,173) ,6.04e-05; (5,173) ,6.04e-05; (6,173) ,6.04e-05; (7,173) ,6.04e-05; (10,173)
,6.04e-05; (11,173) ,6.04e-05; (13,173) ,6.04e-05; (14,173) ,6.04e-05; (22,173) ,6.04e-05; (24,173)
,6.04e-05; (28,173) ,6.04e-05; (33,173) ,6.04e-05; (36,173) ,6.04e-05; (37,173) ,6.04e-05; (38,173)
,6.04e-05; (39,173) ,6.04e-05; (49,173) ,6.04e-05; (64,173) ,6.04e-05; (69,173) ,6.04e-05; (86,173)
,6.04e-05; (119,173) ,6.04e-05; (132,173) ,6.04e-05; (138,173) ,6.04e-05; (143,173) ,6.04e-05;
(2,174) ,0.0383348; (3,174) ,0.0383348; (4,174) ,0.0383348; (5,174) ,0.0383348;
(6,174) ,0.0383348;
(7,174) ,0.0383348; (8,174) ,0.0383348; (9,174) ,0.0383348; (10,174) ,0.0383348;
(11,174) ,0.0383348;
(12,174) ,0.0383348; (13,174) ,0.0383348; (14,174) ,0.0383348; (15,174) ,0.0383348; (16,174)
,0.0383348; (19,174) ,0.0383348; (20,174) ,0.0383348; (21,174) ,0.0383348; (22,174) ,0.0383348;
(23,174) ,0.0383348; (24,174) ,0.0383348; (26,174) ,0.0383348; (27,174) ,0.0383348; (28,174)
,0.0383348; (29,174) ,0.0383348; (30,174) ,0.0383348; (31,174) ,0.0383348; (32,174) ,0.0383348;

,0.0383348; (29,174) ,0.0383348; (30,174) ,0.0383348; (31,174) ,0.0383348; (32,174) ,0.0383348;
(33,174) ,0.0383348; (34,174) ,0.0383348; (35,174) ,0.0383348; (36,174) ,0.0383348; (37,174)
,0.0383348; (38,174) ,0.0383348; (39,174) ,0.0383348; (41,174) ,0.0383348; (42,174) ,0.0383348;
(44,174) ,0.0383348; (46,174) ,0.0383348; (48,174) ,0.0383348; (49,174) ,0.0383348; (50,174)
,0.0383348; (51,174) ,0.0383348; (52,174) ,0.0383348; (54,174) ,0.0383348; (57,174) ,0.0383348;
(63,174) ,0.0383348; (64,174) ,0.0383348; (67,174) ,0.0383348; (69,174) ,0.0383348; (70,174)
,0.0383348; (73,174) ,0.0383348; (75,174) ,0.0383348; (78,174) ,0.0383348; (79,174) ,0.0383348;
(84,174) ,0.0383348; (85,174) ,0.0383348; (86,174) ,0.0383348; (87,174) ,0.0383348; (97,174)
,0.0383348; (107,174) ,0.0383348; (108,174) ,0.0383348; (112,174) ,0.0383348;
(114,174) ,0.0383348;

(115,174) ,0.0383348; (119,174) ,0.0383348; (122,174) ,0.0383348; (132,174) ,0.0383348; (133,174)
,0.0383348; (135,174) ,0.0383348; (136,174) ,0.0383348; (138,174) ,0.0383348; (142,174) ,0.0383348;
(143,174) ,0.0383348; (2,175) ,3.53e-05; (3,175) ,3.53e-05; (4,175) ,3.53e-05; (5,175) ,3.53e-05;
(6,175) ,3.53e-05; (7,175) ,3.53e-05; (8,175) ,3.53e-05; (9,175) ,3.53e-05; (10,175) ,3.53e-05;
(11,175) ,3.53e-05; (12,175) ,3.53e-05; (13,175) ,3.53e-05; (14,175) ,3.53e-05; (15,175) ,3.53e-05;
(16,175) ,3.53e-05; (19,175) ,3.53e-05; (20,175) ,3.53e-05; (21,175) ,3.53e-05; (22,175) ,3.53e-05;
(23,175) ,3.53e-05; (24,175) ,3.53e-05; (26,175) ,3.53e-05; (27,175) ,3.53e-05; (28,175) ,3.53e-05;
(29,175) ,3.53e-05; (30,175) ,3.53e-05; (31,175) ,3.53e-05; (32,175) ,3.53e-05; (33,175) ,3.53e-05;
(34,175) ,3.53e-05; (35,175) ,3.53e-05; (36,175) ,3.53e-05; (37,175) ,3.53e-05; (38,175) ,3.53e-05;
(39,175) ,3.53e-05; (41,175) ,3.53e-05; (42,175) ,3.53e-05; (44,175) ,3.53e-05; (46,175) ,3.53e-05;
(48,175) ,3.53e-05; (49,175) ,3.53e-05; (50,175) ,3.53e-05; (51,175) ,3.53e-05; (52,175) ,3.53e-05;
(54,175) ,3.53e-05; (57,175) ,3.53e-05; (63,175) ,3.53e-05; (64,175) ,3.53e-05; (67,175) ,3.53e-05;
(69,175) ,3.53e-05; (70,175) ,3.53e-05; (73,175) ,3.53e-05; (75,175) ,3.53e-05; (78,175) ,3.53e-05;
(79,175) ,3.53e-05; (84,175) ,3.53e-05; (85,175) ,3.53e-05; (86,175) ,3.53e-05; (87,175) ,3.53e-05;
(97,175) ,3.53e-05; (107,175) ,3.53e-05; (108,175) ,3.53e-05; (112,175) ,3.53e-05; (114,175)
,3.53e-05; (115,175) ,3.53e-05; (119,175) ,3.53e-05; (122,175) ,3.53e-05; (132,175) ,3.53e-05;
(133,175) ,3.53e-05; (135,175) ,3.53e-05; (136,175) ,3.53e-05; (138,175) ,3.53e-05; (142,175)
,3.53e-05; (143,175) ,3.53e-05; (2,177) ,0.0395826; (3,177) ,0.0395826; (4,177) ,0.0395826; (5,177)
,0.0395826; (13,177) ,0.0395826; (28,177) ,0.0395826; (2,178) ,0.0355222; (3,178) ,0.0355222; (4,178)
,0.0355222; (5,178) ,0.0355222; (6,178) ,0.0355222; (8,178) ,0.0355222; (10,178) ,0.0355222; (13,178)
,0.0355222; (14,178) ,0.0355222; (19,178) ,0.0355222; (22,178) ,0.0355222; (24,178) ,0.0355222;
(28,178) ,0.0355222; (30,178) ,0.0355222; (33,178) ,0.0355222; (35,178) ,0.0355222; (36,178)
,0.0355222; (37,178) ,0.0355222; (38,178) ,0.0355222; (39,178) ,0.0355222; (49,178) ,0.0355222;
(52,178) ,0.0355222; (64,178) ,0.0355222; (69,178) ,0.0355222; (86,178) ,0.0355222; (119,178)
,0.0355222; (132,178) ,0.0355222; (138,178) ,0.0355222; (142,178) ,0.0355222; (143,178) ,0.0355222;
(2,180) ,0.035277; (3,180) ,0.035277; (4,180) ,0.035277; (5,180) ,0.035277; (7,180) ,0.035277;
(10,180) ,0.035277; (11,180) ,0.035277; (13,180) ,0.035277; (28,180) ,0.035277; (33,180) ,0.035277;
(36,180) ,0.035277; (37,180) ,0.035277; (38,180) ,0.035277; (69,180) ,0.035277; (86,180) ,0.035277;
(138,180) ,0.035277; (143,180) ,0.035277; (2,181) ,0.0330102; (3,181) ,0.0330102; (4,181) ,0.0330102;
(5,181) ,0.0330102; (6,181) ,0.0330102; (7,181) ,0.0330102; (9,181) ,0.0330102; (10,181) ,0.0330102;
(11,181) ,0.0330102; (12,181) ,0.0330102; (13,181) ,0.0330102; (14,181) ,0.0330102; (15,181)
,0.0330102; (16,181) ,0.0330102; (19,181) ,0.0330102; (21,181) ,0.0330102; (22,181) ,0.0330102;
(23,181) ,0.0330102; (24,181) ,0.0330102; (26,181) ,0.0330102; (28,181) ,0.0330102; (29,181)
,0.0330102; (30,181) ,0.0330102; (31,181) ,0.0330102; (32,181) ,0.0330102; (33,181) ,0.0330102;

(35,181) ,0.0330102; (36,181) ,0.0330102; (37,181) ,0.0330102; (38,181) ,0.0330102; (39,181) ,0.0330102; (41,181) ,0.0330102; (42,181) ,0.0330102; (46,181) ,0.0330102; (49,181) ,0.0330102; (50,181) ,0.0330102; (52,181) ,0.0330102; (54,181) ,0.0330102; (57,181) ,0.0330102; (64,181) ,0.0330102; (67,181) ,0.0330102; (69,181) ,0.0330102; (70,181) ,0.0330102; (75,181) ,0.0330102; (86,181) ,0.0330102; (97,181) ,0.0330102; (107,181) ,0.0330102; (108,181) ,0.0330102; (114,181)
,0.0330102; (115,181) ,0.0330102; (119,181) ,0.0330102; (133,181) ,0.0330102; (138,181) ,0.0330102;
(142,181) ,0.0330102; (143,181) ,0.0330102; (2,183) ,0.0361979; (3,183) ,0.0361979; (4,183) ,0.0361979; (5,183) ,0.0361979; (6,183) ,0.0361979; (7,183) ,0.0361979; (14,183) ,0.0361979; (22,183)
,0.0361979; (24,183) ,0.0361979; (28,183) ,0.0361979; (33,183) ,0.0361979; (36,183) ,0.0361979; (38,183) ,0.0361979; (39,183) ,0.0361979; (49,183) ,0.0361979; (64,183) ,0.0361979; (86,183) ,0.0361979; (119,183) ,0.0361979; (132,183) ,0.0361979; (138,183) ,0.0361979; (2,184) ,0.036786;
(3,184) ,0.036786; (4,184) ,0.036786; (5,184) ,0.036786; (6,184) ,0.036786; (7,184) ,0.036786; (8,184) ,0.036786; (9,184) ,0.036786; (10,184) ,0.036786; (12,184) ,0.036786; (13,184) ,0.036786;
(14,184) ,0.036786; (15,184) ,0.036786; (16,184) ,0.036786; (20,184) ,0.036786; (21,184) ,0.036786;
(22,184) ,0.036786; (24,184) ,0.036786; (26,184) ,0.036786; (27,184) ,0.036786; (28,184) ,0.036786;
(29,184) ,0.036786; (30,184) ,0.036786; (32,184) ,0.036786; (33,184) ,0.036786; (34,184) ,0.036786;
(35,184) ,0.036786; (36,184) ,0.036786; (37,184) ,0.036786; (38,184) ,0.036786; (39,184) ,0.036786;
(42,184) ,0.036786; (44,184) ,0.036786; (46,184) ,0.036786; (48,184) ,0.036786; (49,184) ,0.036786;
(50,184) ,0.036786; (51,184) ,0.036786; (52,184) ,0.036786; (54,184) ,0.036786; (63,184) ,0.036786;
(64,184) ,0.036786; (67,184) ,0.036786; (69,184) ,0.036786; (70,184) ,0.036786; (73,184) ,0.036786;
(75,184) ,0.036786; (78,184) ,0.036786; (79,184) ,0.036786; (84,184) ,0.036786; (85,184) ,0.036786;
(86,184) ,0.036786; (87,184) ,0.036786; (107,184) ,0.036786; (108,184) ,0.036786; (112,184) ,0.036786; (114,184) ,0.036786; (119,184) ,0.036786; (122,184) ,0.036786; (132,184) ,0.036786; (135,184) ,0.036786; (136,184) ,0.036786; (138,184) ,0.036786; (142,184) ,0.036786; (143,184) ,0.036786; (2,186) ,0.0388301; (3,186) ,0.0388301; (4,186) ,0.0388301; (5,186) ,0.0388301; (6,186)

,0.036786; (2,186) ,0.0388301; (3,186) ,0.0388301; (4,186) ,0.0388301; (5,186) ,0.0388301; (6,186)
,0.0388301; (36,186) ,0.0388301; (138,186) ,0.0388301; (2,187) ,0.0361996; (3,187) ,0.0361996; (4,187) ,0.0361996; (5,187) ,0.0361996; (6,187) ,0.0361996; (7,187) ,0.0361996;

(11,187) ,0.0361996;
(12,187) ,0.0361996; (13,187) ,0.0361996; (14,187) ,0.0361996; (15,187) ,0.0361996; (21,187) ,0.0361996; (24,187) ,0.0361996; (26,187) ,0.0361996; (28,187) ,0.0361996; (29,187) ,0.0361996; (32,187) ,0.0361996; (33,187) ,0.0361996; (35,187) ,0.0361996; (36,187) ,0.0361996; (37,187) ,0.0361996; (38,187) ,0.0361996; (42,187) ,0.0361996; (46,187) ,0.0361996; (49,187) ,0.0361996; (50,187) ,0.0361996; (52,187) ,0.0361996; (54,187) ,0.0361996; (69,187) ,0.0361996; (70,187) ,0.0361996; (84,187) ,0.0361996; (86,187) ,0.0361996; (119,187) ,0.0361996; (138,187) ,0.0361996;
(142,187) ,0.0361996; (143,187) ,0.0361996;

Step 3: data partitioning, the trainingData proportion: 0.7, the data distribution: sum - + data 142 41 101 train 98 28 70 test 44 13 31

Step 4: data preprocessing. the selected method: Standardization((fi-u)/std).

step 5: feature selection, the selective method: Lasso.You will find a best lambda by using cross validation. bestLoglamda: 1.2. show error-lambda graph. show coeffcients-lambda graph. . remained
feature number: 14. remained feature name order: GLCMEntropy_AllDirection_offset4. GLCMEntropy_AllDirection_offset7. GLCMEntropy_angle0_offset1. GLCMEntropy_angle0_offset4.
GLCMEntropy_angle0_offset7. GLCMEntropy_angle135_offset1. GLCMEntropy_angle135_offset4.
GLCMEntropy_angle135_offset7. GLCMEntropy_angle45_offset1. GLCMEntropy_angle45_offset4.
GLCMEntropy_angle90_offset1. GLCMEntropy_angle90_offset4. HaraEntroy. the trainingData proportion:
0.7, the data distribution: sum - + data 142 41 101 train 98 28 70 test 44 13 31 the feature number
is: 14

step 5: feature selection, the selective method: Correlation Analysis. threshold value: 0.9.correlation method: spearman. . remained feature number: 2. empirical feature number: 9. remained
feature name order: GLCMEntropy_AllDirection_offset1. GLCMEntropy_angle135_offset7. the trainingData
proportion: 0.7, the data distribution: sum - + data 142 41 101 train 98 28 70 test 44 13 31 the feature number is: 2

step 7: machine learning, the selected method:SVM, logistic regression,RF
