# Supplementary material for: Predicting the Grade of Prostate Cancer Based on a Biparametric MRI Radiomics Signature
Source: Contrast Media Mol Imaging. 2021 Dec 23;2021:7830909. doi: 10.1155/2021/7830909 (PMC8718299; doi:10.1155/2021/7830909)
Supplement: Supplementary Materials — The supplementary file contains the supplementary information about the original data for radiomics (Supplementary Materials and Supplement Tables 1–6). [file 7830909.f1.zip › 7830909.f1/Supplementary Materials(tables).pdf]

Table 1 PI-RADS V2.1 Assessment for T2WI

Score	Peripheral Zone (PZ)
1	Uniform hyperintense signal intensity(normal)
2	Linear or wedge-shaped hypointensity or diffuse mild hypointensity, usually indistinct margin
3	Heterogeneous signal intensity or non-circumscribed, rounded, moderate hypointensity<br>Includes others that do not qualify as 2, 4, or 5
4	Circumscribed, homogenous moderate hypointense focus/mass confined to prostate and <1.5 cm in greatest dimension
5	Same as 4 but ≥1.5cm in greatest dimension or definite extraprostatic extension/invasive behavior
**Score**	**Transition Zone (TZ)**
1	Normal appearing TZ (rare) or a round, completely encapsulated nodule.("typical nodule")
2	A mostly encapsulated nodule OR a homogeneous circumscribed nodule without encapsulation. ("atypical nodule") OR a homogeneous mildly hypointense area between nodules
3	Heterogeneous signal intensity with obscured margins<br>Includes others that do not qualify as 2, 4, or 5
4	Lenticular or non-circumscribed, homogeneous, moderately hypointense, and <1.5 cm in greatest dimension
5	Same as 4, but ≥1.5cm in greatest dimension or definite extraprostatic extension/invasive behavior

Table 2 PI-RADS V2.1 Assessment of DWI

Score	Peripheral Zone (PZ) or Transition Zone (TZ)
1	No abnormality (i.e., normal) on ADC and high b-value DWI
2	Linear/wedge shaped hypointense on ADC and/or linear/wedge shaped hyperintense on high b-value DWI
3	Focal (discrete and different from the background) hypointense on ADC and/or focal hyperintense on high b-value DWI;<br>may be markedly hypointense on ADC or markedly hyperintense on high b-value DWI, but not both.
4	Focal markedly hypontense on ADC and markedly hyperintense on high b-value DWI;<br><1.5cm in greatest dimension
5	Same as 4 but ≥1.5cm in greatest dimension or definite extraprostatic extension/invasive behavior

Table 3 PI-RADS V2.1 Assessment for DCE

Score	Peripheral Zone (PZ) or Transition Zone (TZ)
(-)	no early or contemporaneous enhancement;or diffuse multifocal enhancement NOT corresponding to a focal finding on T2W and/or DWI or focal enhancement corresponding to a lesion demonstrating features of BPH on T2WI (including features of extruded BPH in the PZ)
(+)	focal, and; earlier than or contemporaneously with enhancement of adjacent normal prostatic tissues, and; corresponds to suspicious finding on T2W and/or DWI

Table 4 PI-RADS V2.1 Assessment for Peripheral Zone (PZ)

DWI	T2W	DCE	PIRADS
1	Any	Any	1
2	Any	Any	2
3	Any	-	3
		+	4
4	Any	Any	4
5	Any	Any	5

Table 5 PI-RADS Assessment for Transition Zone (TZ)

T2WI	DWI	DCE	PIRADS
1	Any	Any	1
2	≤3	Any	2
	≥4	Any	3
3	≤4	Any	3
	5	Any	4
4	Any	Any	4
5	Any	Any	5

Table 6 The scores for each case based on PI-RADS V2.1 scale of Radiologist A and B

Patient	Radiologist A	Radiologist B
1	4	4
2	2	3
3	5	4
4	4	4
5	3	3
6	3	2
7	5	5
8	4	4
9	1	2
10	4	4
11	4	4

12	5	5
13	3	4
14	1	2
15	4	4
16	5	4
17	4	4
18	4	4
19	2	2
20	5	5
21	4	4
22	3	3
23	4	4
24	4	4
25	4	4
26	5	5
27	1	2
28	4	4
29	4	4
30	4	4
31	4	4
32	2	4
33	4	4
34	3	3
35	4	4
36	3	3
37	5	4
38	5	4
39	5	5
40	4	4
41	4	4
42	3	3
43	5	5
44	4	4
45	4	4
46	5	5
47	4	4
48	5	4
49	4	4
50	4	4
51	4	4
52	5	5
53	4	4

54	5	5
55	5	5
56	5	5
57	5	5
58	5	5
59	5	5
60	5	5
61	5	5
62	4	4
63	1	2
64	5	5
65	5	5
66	2	2
67	5	5
68	5	4
69	4	4
70	5	5
71	5	5
72	4	5
73	5	5
74	5	5
75	5	5
76	5	5
77	5	5
78	4	4
79	5	5
80	5	5
81	5	5
82	4	4
83	4	4
84	5	5
85	4	4
86	5	5
87	5	5
88	5	5
89	5	5
90	5	5
91	5	5
92	5	5
93	5	5
94	5	5
95	5	5

96	4	4
97	5	5
98	5	5
99	5	5
100	5	5
101	5	5
102	5	5
103	5	5
104	5	5
105	5	5
106	5	5
107	5	5
108	5	5
109	5	5
110	5	5
111	4	4
112	5	5
113	4	4
114	4	4
115	5	5
116	5	5
117	5	5
118	4	4
119	5	5
120	4	4
121	3	4
122	5	5
123	5	5
124	5	5
125	5	5
126	5	5
127	4	5
128	5	5
129	5	5
130	5	5
131	4	4
132	5	5
133	5	5
134	4	5
135	5	5
136	5	5
137	4	4

138	5	5
139	5	5
140	5	5
141	3	3
142	4	4
